# A fully analytical integration of properties over the 3D volume of the *β* sphere in topological atoms

**DOI:** 10.1002/jcc.25158

**Published:** 2018-01-10

**Authors:** Paul L. A. Popelier

**Affiliations:** ^1^ Manchester Institute of Biotechnology (MIB), University of Manchester, 131 Princess Street, Manchester M1 7DN Great Britain; ^2^ School of Chemistry University of Manchester, Oxford Road Manchester M13 9PL Great Britain

**Keywords:** QTAIM, quantum chemical topology, integration, spherical Bessel function, beta sphere

## Abstract

Atomic multipole moments associated with a spherical volume fully residing within a topological atom (i.e., the *β* sphere) can be obtained analytically. Such an integration is thus free of quadrature grids. A general formula for an arbitrary rank spherical harmonic multipole moment is derived, for an electron density comprising Gaussian primitives of arbitrary angular momentum. The closed expressions derived here are also sufficient to calculate the electrostatic potential, the two types of kinetic energy, as well as the potential energy between atoms. Some integrals have not been solved explicitly before but through recursion and substitution are broken down to more elementary listed integrals. The proposed method is based on a central formula that shifts Gaussian primitives from one center to another, which can be derived from the well‐known plane‐wave expansion (or Rayleigh equation). © 2018 The Authors. Journal of Computational Chemistry Published by Wiley Periodicals, Inc.

## Introduction

Quantum chemical topology (QCT),^1^ pioneered^2^ by the research group of the late Richard Bader, has carved out a space among non‐topological methods in extracting insight from wave functions, both in chemistry (including metal‐metal interactions^3^) and solid state physics (including high‐resolution X‐ray crystallography^4^). This imaginative approach started, in quantum chemistry, as (the Quantum Theory of) Atoms in Molecules^5^ (QT)(AIM) in the early 1970s. After adopting the mathematical language of dynamical systems with an eye on analyzing the electron density, QTAIM proposed a parameter‐free definition of an atom and a chemical bond (the latter continuing to be a topic of contemporary research). The concepts of dynamical systems (e.g., separatrix, critical point, basin, gradient path) have been applied with success to quantum mechanical density functions, other than the electron density, such as the Laplacian of the electron density, the nuclear potential, the electron localization function, the electron localizability indicator, the virial field, to name a few (references and more details see both the Introduction and Box 8.1 in Ref. 6). A detailed history of QTAIM and its QCT follow up has been given elsewhere.^6^, ^7^, ^8^

Although sometimes still mistaken^9^ for yet another population analysis, QTAIM offers a good number of important atomic properties other than an atomic charge, which all derive from a single universal formula. This formula is the 3D integration of a relevant integrand over the volume of the topological atom, and can yield atomic (kinetic) energy, volume, electrostatic potential, and multipole moments, to name a few common ones. The existence of a single formula that delivers atomic properties in a consistent way should at least be emphasized and perhaps even celebrated. Indeed, this is not true for other methods: for example, there is no Mulliken volume or Hirshfeld volume; similarly, there is no van der Waals charge (as opposed to volume or radius). Better controlling the atomic integration over a spherical volume that resides completely within a topological atom (see below) is a sign of progress.

An important development within the QCT framework is that of an energy partitioning method called interacting quantum atoms (IQA),^10^ which is based on earlier work.^11^ In spite of its great computational cost, IQA is increasingly applied to a wide variety of chemical phenomena, non‐exhaustively ranging from hydrogen bond cooperativity^12^ over metal carbonyl bonds,^13^ halogen bonds,^14^ Zn‐complexes,^15^ excited states,^16^ and congested molecules,^17^ to torsional energy barriers in peptides.^18^ IQA hereby avoids the pitfalls,^19^ both conceptual and numerical, of older non‐topological energy partitioning schemes such as EDA and SAPT. The surge of IQA has been made possible by improved algorithms (e.g., parallelization and parameter fine‐tuning) such as those found in the program^20^ AIMAll.

Still, the computational challenge of the topological partitioning remains, compared to cheaper non‐topological methods. This is why research that improves algorithms to integrate topological atoms should continue. This article focuses on the elimination of integration quadrature, but only inside the so‐called *β* sphere,^21^ which is the largest sphere that is completely contained within the topological atom at whose nucleus it is centered. In practice, the *β* sphere is just a sphere with an adequately large radius, typically ∼90% of the distance between the nucleus and the nearest bond critical point. Numerical volume integration over the whole topological atom does not require a *β* sphere, in principle. Indeed, the radial quadrature can be performed by a single interval stretching from the nucleus to the edge of the atom. However, the very high values of electron density near the nucleus make it numerically advantageous to keep the *β* sphere, and to work with two radial integration intervals, one inside the *β* sphere and one outside, each with its own quadrature grid. Heavy elements (fourth period and beyond) benefit from even more than two radial integration intervals. In 2011, a fully analytical 3D volume integration over the *β*‐sphere was achieved for the first time.^22^ Here, we propose an alternative analytical integration, which not only avoids the tedious axes rotations occurring in the former integration procedure but also generalizes much better to arbitrary multipole moments, and enables controlled elimination of Gaussian primitive functions far from the nucleus of the atom being integrated.

The Introduction to the 2011 paper contains a very detailed and rather exhaustive history at the time of topological integration algorithms, which will not be repeated here. Instead, we expeditiously review the literature, now extending it with the period 2012–2017, and starting with the first integrations over topological atoms, carried out^23^ already in 1973 but for linear molecules only. In 1981, the first computer program called OMEGA^21^ enabled the integration of topological atoms in any polyatomic molecule, followed by a completely different program^24^ a year later, called PROAIM. The Bader group gathered these programs, together with critical point location software, into the AIMPAC suite. With the help of collaborators, the main author of the atomic integration code, mathematician Biegler‐König, resurrected and perfected^25^, ^26^, ^27^ his effort of the 1980s, leading to the AIM2000 code. Meanwhile, in the 1990s, the program MORPHY^28^ appeared as the first integration code^29^ written independently from the AIMPAC suite. MORPHY, also for the first time combined all AIMPAC functionality into a single program. In 1997, AIMAll emerged from an extensive AIMPAC modification, and is now a popular and fast QCT code. Independent code, also developed^30^ in the 1990s, was implemented in the program GAUSSIAN but later withdrawn from it. In 1997, a research group in Oviedo (Spain, USE) created an independently written topological code called CRITIC,^31^ which is devoted to solid state systems, and later honed it to CRITIC2.^32^ In the early 2000s, they also wrote PROMOLDEN, a code specialized in molecular rather than solid state electron densities, and focusing on IQA. The QTREE method was also invented^33^ in Oviedo, and was based on the OCTREE algorithm^34^ proposed eight years earlier. The Electron Localization Function, a popular QCT tool^35^ to extract chemical information from wave functions, triggered the independent development of the topological code TopMoD.^36^ Gatti's TOPOND code^37^ determines the topology of an analytically expressed Hartree‐Fock or DFT wavefunction obtained by the program CRYSTAL.^38^ The high‐resolution X‐ray crystallography community also created its own algorithms early on such as that of TOPXD,^39^ NEWPROP,^40^ or WINXPRO.^41^ However, a topological analysis can also be carried out using grids,^42^, ^43^ such as the InteGriTy package.^44^ Grid methods are also applied in the context of computed electron densities.^45^, ^46^, ^47^ Two more algorithms are based on different principles, such as the “elastic sheet” method^48^ and a finite element method.^49^ In contrast, recent developments^50^, ^51^ turned back to the original triangulation method of Biegler‐König. Meanwhile, in the 2010s, the Chinese program Multiwfn^52^ (multifunctional wavefunction analyzer) emerged from a fast catch‐up exercise consisting of rewriting a large collection of pre‐existing algorithms developed over years by other groups. Multiwfn has an impressive functionality, encompassing both topological and non‐topological methods, but encourages crude parameter settings, and typically implements the simplest (i.e., mathematically elementary) algorithms.

In this article, we focus on the involved mathematics of deriving closed formulae for the *β* sphere's contribution to atomic multipole moments of arbitrary spherical harmonic rank, the two types of kinetic energy (K and G), the electrostatic potential and the potential energy between topological atoms. To keep the Method Section to the point, much material has been siphoned off to the Supporting Information. Due to the mathematical complexity of the analytical approach, the strategy and all details are reported in this article, while an implementation will follow in a future software‐oriented publication.

## Method

Imagine a sphere with a given radius *β*, centered at a given nuclear position. In the context of topological atoms, this sphere is called the *β* sphere. This is the largest sphere that completely fits within a topological atom. Whichever the shape of this atom's interatomic surfaces, *β* must be smaller than the distance between the nucleus and the closest‐by bond critical point.

The first aim is to calculate, by analytical integration, the atomic multipole moments *Q_LM_* associated with the volume of the *β* sphere only. The integrand of the integral leading to *Q_LM_*(*β*) is the product of the electron density *ρ* and a regular spherical harmonic *R_L,M_* centered on the center of atomic integration with position vector **R**_*i*__,_

(1) $$Q_{LM}(\beta )=\int_{\beta \;\;\text{sphere}}dr\;\rho (r)\;R_{L,M}(r-R_{i})$$

where the radius *β* acts as a parameter and where the regular spherical (or solid) harmonic *R_L,M_* is defined by eq. (2),

(2) $$R_{L,M}(r,\theta ,\varphi )=\sqrt{\frac{4\pi }{2L+1}}\;r^{L}Y_{L,M}(\theta ,\varphi )$$

Here *r, θ*, and *φ* represent spherical coordinates centered on **R**_*i*_, which typically is the nucleus of (topological) atom Ω and *Y_L,M_* represents a spherical harmonic, defined using the so‐called “quantum mechanics convention” and including the Condon‐Shortley factor.

Three comments are in order here. First, the uppercase indices L and M should not be confused with the lowercase indices ℓ and m, which will appear in formulae below. The regular spherical harmonics share their mathematical shape with the familiar s, p, d, f orbitals, or orbitals of higher rank. Second, eq. (2) defines the *R_L,M_* functions as complex (for details see Part A in the Supporting Information). It is best to work with complex *R_L,M_* functions during a derivation and, if necessary, convert them to real functions only at the end. Third, there is a local frame centered on a given nucleus, which determines the orientation of *R_LM_* in space. This local frame is parallel to the global frame, which renders the local and global frames identical but for a translation. Earlier work^22^ shows that one can make the nuclear position of the *β* sphere coincide with the origin of the global frame, without loss of generality.

The electron density appearing in eq. (1) is expanded in Gaussian primitives (denoted *G*(**r – R**)) of arbitrary angular momentum and centered at position **R**,

(3) $$\begin{array}{l} \rho (r)=\sum_{p=1}^{n_{\text{MO}}}n_{p}\psi_{p}^{2}(r)=\;\sum_{i=1}^{n_{\text{MO}}}n_{p}\left(\sum_{j=1}^{n_{G}}c_{jp}G_{j}(r-R_{j})\right)\left(\sum_{k=1}^{n_{G}}c_{kp}G_{k}(r-R_{k})\right)\\ \;\;=\sum_{p=1}^{n_{\text{MO}}}\sum_{j=1}^{n_{G}}\sum_{k=1}^{n_{G}}n_{p}c_{kp}c_{jp}G_{j}(r-R_{j})G_{k}(r-R_{k}) \end{array}$$

where *n*_MO_ signifies the number of molecular orbitals, *n*_G_ is the number of Gaussian primitives used to expand each MO in, and *c* are the LCAO coefficients, which include the normalization factors of the Gaussian primitives. This notation makes clear that a Gaussian primitive is centered on either nuclear position **R**_*j*_ or **R**_*k*_. Note that **R**_*j*_ may or may not coincide with the center of atomic integration **R**_*i*_. The same is true for **R**_*k*_, independently of whether **R**_*j*_ coincides with **R**_*i*_.

A brief digression points out that, in a quadrature scheme, it is possible to evaluate a molecular orbital on its own, as part of the integration procedure calculating the integral of eq. (1). However, in an analytical integration, one cannot do this and one must instead consider the whole electron density in the integrand in eq. (1). This point is elaborated in *Appendix A* of the Supporting Information of Ref. 22.

Using the Gaussian product theorem, the product of any two Gaussians centered on **R**_*j*_ and **R**_*k*_ can be written as a single Gaussian, centered on **R**_*jk*_. Ignoring the angular part of the Gaussian primitives, the product theorem is formally written as

(4) $$G_{j}(r-R_{j})G_{k}(r-R_{k})=\exp(-\alpha_{j}{|r-R_{j}|}^{2})\exp(-\alpha_{k}{|r-R_{k}|}^{2})=K_{jk}\exp(-\alpha_{jk}{|r-R_{jk}|}^{2})=G_{jk}(r-R_{jk})$$

The new Gaussian exponent *α_jk_*, the new center **R**_*jk*_ and prefactor *K_jk_* are also easily calculated,

(5) $$\alpha_{jk}{}_{}=\alpha_{j}{}_{}+\alpha_{k},\;R_{jk}=\frac{\alpha_{j}R_{j}+\alpha_{k}R_{k}}{\alpha_{j}+\alpha_{k}},\;K_{jk}=\exp(-\frac{\alpha_{j}\alpha_{k}}{\alpha_{j}+\alpha_{k}}{|R_{j}-R_{k}|}^{2})$$

Substitution of eq. (3) into eq. (1) leads to eq. (6),

(6) $$Q_{LM}(\beta )=\;\sum_{p=1}^{n_{\text{MO}}}n_{p}\sum_{j=1}^{n_{G}}\sum_{k=1}^{n_{G}}c_{kp}\;c_{jp}\int_{\beta }^{}dr\;R_{L,M}(r-R_{i})\;G_{jk}(r-R_{jk})$$

The integration problem is then confined to the contribution from a single Gaussian to a given multipole moment and denoted *Q_LM, jk_*(*β*), which is centered on a site that is, in principle, different to the site **R**_*j*_ at which the multipole moment is centered,

(7) $$Q_{LM,\;jk}(\beta )=\;\int_{\beta }^{}dr\;R_{L,M}(r-R_{i})\;G_{jk}(r-R_{jk})$$

Without loss of generality one can position the atomic integration center **R**_*i*_ at the global origin, which is equivalent to translate the whole relative configuration of vectors **r**, **R**_*j*_, **R**_*k*_, and **R**_*jk*_ to the global origin.

**Figure 1:**
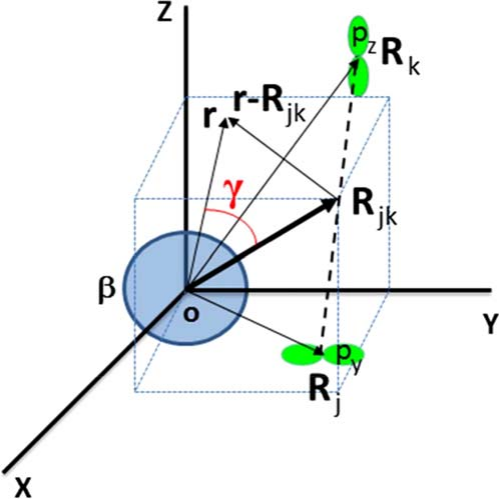
Schematic representation of the vectors involved in eqs. (3), (6), and (7). The atom to be integrated is centered at the origin **o** of the global frame, which provides coordinates for all vectors involved. Two arbitrary Gaussian primitives, a *p_y_* and a *p_z_* function, are respectively centered at **R**_j_ and **R**_k_. The product of these Gaussian primitives is centered at **R**_jk_. The position vector **r** describes the electron density contributing to the atomic (volume) integral. The angle *γ* is pivotal in the separation of the variables. [Color figure can be viewed at wileyonlinelibrary.com]

Figure 1 illustrates all vectors introduced so far, and how they relate to one another geometrically, for an arbitrary pair of Gaussian primitives centered on **R**_*j*_ and **R**_*k*_, and contributing to the atomic integral calculated in eq. (7). Figure 1 illustrates the case of two Gaussian p‐type primitives, p_*y*_ and p_*z*__,_ making a contribution to *ρ*(**r**) and hence to *Q_LM_*.

For the general product of two Gaussian primitives of arbitrary angular momentum, eq. (7) becomes

(8) $$Q_{LM,jk}(\beta )=\;K_{jk}\int_{\beta }^{}dr\;R_{L,M}(r)\;\exp(-\alpha_{jk}{|r-R_{jk}|}^{2}){\left(x-X_{j}\right)}^{{\bar{l}}_{j}}{\left(y-Y_{j}\right)}^{{\bar{m}}_{j}}{\left(z-Z_{j}\right)}^{{\bar{n}}_{j}}{\left(x-X_{k}\right)}^{{\bar{l}}_{k}}{\left(y-Y_{k}\right)}^{{\bar{m}}_{k}}{\left(z-Z_{k}\right)}^{{\bar{n}}_{k}}$$

where the indices *l, j*, and *k* with superscript bars mark the angular momenta. One can use the binomial expression to work out each of the three products in eq. (8), one product in *x*, one in *y* and one in *z*. For example, focusing on *x* only, one obtains eq. (9),

(9) $${\left(x-X_{j}\right)}^{{\bar{l}}_{j}}{\left(x-X_{k}\right)}^{{\bar{l}}_{k}}=\sum_{\lambda =0}^{N_{\lambda }}f_{\lambda x}(X_{j},X_{k})x^{\lambda }$$

where the function *f_λx_* provides the coefficients corresponding to the power *λ* = *r* + *s* in *x,* which parametrically depend on the position of the centers (*X_j_*, *X_k_*) of the Gaussian primitives and their angular momenta (the various powers of *X_j_* and *X_k_*). We can then deduce from eq. (9) the two lowest powers of *λ* (0 and 1),

(10) $$\begin{array}{l} f_{0x}(X_{j},X_{k})={\left(-X_{j}\right)}^{{\bar{l}}_{j}}{\left(-X_{k}\right)}^{{\bar{l}}_{k}}\\ f_{1x}(X_{j},X_{k})={\bar{l}}_{k}{\left(-X_{j}\right)}^{{\bar{l}}_{j}}{\left(-X_{k}\right)}^{{\bar{l}}_{k}-1}+{\bar{l}}_{j}{\left(-X_{j}\right)}^{{\bar{l}}_{j}-1}{\left(-X_{k}\right)}^{{\bar{l}}_{k}} \end{array}$$

while Part B of the Supporting Information explicitly lists *f*_2_ *_x_* and *f*_3__*x*_. The functions *f_n_* are polynomials in the components of the position vectors **R**_*j*_ and **R**_*k*_, which are expressed with respect to the global axis system (which is translated to the center of integration, for each atom in turn, as discussed above).

Equation (8) can then be rearranged as

(11) $$\begin{array}{l} Q_{LM,jk}(\beta )=\;K_{jk}\int_{\beta }^{}dr\;R_{L,M}(r)\;\exp(-\alpha_{jk}{|r-R_{jk}|}^{2})\left(\sum_{\lambda =0}^{}f_{\lambda x}(X_{j},X_{k})x^{\lambda }\right)\left(\sum_{\mu =0}^{}f_{\mu y}(Y_{j},Y_{k})y^{\mu }\right)\left(\sum_{\nu =0}^{}f_{\nu z}(Z_{j},Z_{k})z^{\nu }\right)\\ \;\;\;\;\;=K_{jk}\sum_{\lambda =0}^{}\sum_{\mu =0}^{}\sum_{\nu =0}^{}f_{\lambda x}f_{\mu y}f_{\nu z}\int_{\beta }^{}dr\;R_{LM}(r)\;\exp(-\alpha_{jk}{|r-R_{jk}|}^{2})x^{\lambda }y^{\mu }z^{\nu } \end{array}$$

where *λ* is the integer power of the coordinate *x, μ* that of *y,* and *ν* that of *z*. Part B of the Supporting Information also applies eqs. (11) and (10) to the specific case illustrated in Figure 1, which shows two different p‐type Gaussian primitives (p_*y*_ and p_*z*_) contributing to *Q_LM_*_,__*jk*_.

To work out eq. (11) analytically, we recognize that *R_LM_* and the product Gaussian *G_jk_* have a different origin: the former is centered at the global origin **o** while the latter is centered at **R**_*jk*_. The integral of eq. (11) is naturally carried out in spherical coordinates centered at **o** because the electron density is essentially spherical, even for an atom inside a molecule. In other words, the deviations from sphericity are too small to abandon spherical coordinates in favor of alternative but inappropriate coordinates, such as Cartesian coordinates. The challenge is therefore to re‐express *G_jk_* in terms of spherical coordinates centered at **o**. This can be achieved using a key equation proposed before,^53^

(12) $$\exp(r_{1}•r_{2})=\sum_{\ell =0}^{\infty }(2\ell +1)\;i_{\ell }(r_{1}r_{2})P_{\ell }(\cos\gamma )$$

where *i*_ℓ_(*z*) is the modified spherical Bessel function of integer order ℓ, and *r*_1_ and *r*_2_ are the respective magnitudes of vector **r**_1_ and **r**_2_, while P_ℓ_ is the Legendre function of order ℓ and *γ* is the angle between these two vectors. Six remarks are in order here. First, eq. (12) can be derived from the well‐known plane‐wave expansion (or Rayleigh equation) as demonstrated in Part C of the Supporting Information. Second, no convergence condition accompanying this series expansion is reported. Third, the sum over ℓ needs to be truncated to a finite integer, which offers an opportunity to make the integral evaluation (see below) more efficient. Fourth, on application in the current context, which vector is **r**_1_ and which is **r**_2_ is not important because eq. (12) remains valid if these vectors are swapped. Fifth, the expansion of eq. (12) is pointless if either **r**_1_ or **r**_2_ vanishes. In that case, we trivially recover that 1 = 1. Indeed, the only surviving term in the sum is that of ℓ = 0, and *i*_0_(0) = 1 and *P*_0_(*x*) = 1 (even when *γ* is indeterminate, as is the case here, with an undefined angle *γ* resulting between a null vector and non‐null vector). Sixth and finally, the argument of an exponential function must be dimensionless and hence, if **r_1_** has the dimension of length then **r_2_** must have the dimension of reciprocal length, which is the case if it were a wave factor (as indeed it is in the Rayleigh equation).

To make use of eq. (12), the exponential function's argument in eq. (11) needs to be rewritten. This is done in eq. (13),

(13) $$\exp(-\alpha_{jk}{|r-R_{jk}|}^{2})=\exp\left(-\alpha_{jk}(r^{2}+R_{jk}^{2})\right)\exp(2\alpha_{jk}R_{jk}•r)$$

such that eq. (12) applies to the second factor on the right‐hand side of eq. (13), setting **r**_1_ = 2*α_jk_* **R**_*jk*_ and **r**_2_ = **r** where |**R**_*jk*_| = *R_jk_* and |**r**| = *r*. In terms of dimensionality (see 6th remark just above), the dimension of *α* is reciprocal length squared, and hence, the argument of the exponential in eq. (13) is again dimensionless. Substitution of eqs. (12) and (13) into eq. (11), and dropping the by now tedious indices *j* and *k*, one obtains eq. (14),

(14) $$Q_{LM}(\beta )=K\exp(-\alpha R_{}^{2})\sum_{\lambda =0}^{}\sum_{\mu =0}^{}\sum_{\nu =0}^{}f_{\lambda x}f_{\mu y}f_{\nu z}\int_{\beta }^{}dr\;R_{LM}(r)\exp(-\alpha r^{2})\sum_{\ell =0}^{\infty }\left(2\ell +1)i_{\ell }(2\alpha Rr)P_{\ell }(\cos\gamma \right)\;x^{\lambda }y^{\mu }z^{\nu }$$

Two remarks must be made. If either **R** = **0** (center of product Gaussian coincides with the center of integration) or **r** = **0** (lower boundary of volume integration), then the expansion of eq. (12) becomes trivial but it still holds. From the latter statement, it follows that there is no need to treat the case of **R** = **0** separately, at least at this stage. Later in the derivation a further comment on **R** = **0** will be needed. In summary, although the case of **R** = **0** can be solved directly using spherical coordinates centered on the global origin in eq. (11) we proceed anyway with the expansion that is eq. (12). Second, the quantities *R, K, α*, and *f* in eq. (14) are parameters, depending on the fixed characteristics of the product Gaussian. The volume integral in eq. (14) is best described via spherical coordinates, denoted *r*, *θ*, and *φ*, and centered at the global origin **o** (see Fig. 1). The quantity *γ* is the angle between **r** and **R** and hence is a mixture of a parameter (**R**) and a variable (**r**). We aim to separate all variables from parameters, so the *P*_l_(cos *γ*) factor needs to be expanded. The well‐known addition theorem involving two spherical harmonics, given in eq. (15), achieves this aim,

(15) $$P_{\ell }(\cos\gamma )=\frac{4\pi }{2\ell +1}\sum_{m=-\ell }^{\ell }Y_{\ell ,m}{}^{*}(\theta ,\varphi )Y_{\ell ,m}^{}(\theta_{R},\varphi_{R})$$

where vectors **r** and **R** are, respectively, described by spherical coordinates (*r*, *θ*, *φ*) and (*R*, *θ*_R_, *φ*_R_), which are both centered on **o**. The choice as to which variables describe the complex conjugate spherical harmonic is steered by the application of an advantageous orthonormalization relationship discussed below, the special case where *λ* = *ν* = *μ* = 0. Substituting eq. (15) into eq. (14), and rearranging delivers

(16) $$Q_{LM}(\beta )=4\pi K\exp(-\alpha R_{}^{2})\sum_{\lambda =0}^{}\sum_{\mu =0}^{}\sum_{\nu =0}^{}f_{\lambda x}f_{\mu y}f_{\nu z}\int_{\beta }^{}dr\;R_{LM}(r)\exp(-\alpha r^{2})\sum_{\ell =0}^{\infty }i_{\ell }(2\alpha Rr)\sum_{m=-\ell }^{\ell }Y_{\ell ,m}{}^{*}(\theta ,\varphi )Y_{\ell ,m}^{}(\theta_{R},\varphi_{R})\;x^{\lambda }y^{\mu }z^{\nu }$$

Substituting eq. (2) into eq. (16) and making integration variables explicit yields,

(17) $$\begin{array}{l} Q_{LM}(\beta )=\;\;4\pi \sqrt{\frac{4\pi }{2L+1}}\;\;K\exp(-\alpha R_{}^{2})\sum_{\lambda =0}^{}\sum_{\mu =0}^{}\sum_{\nu =0}^{}f_{\lambda x}f_{\mu y}f_{\nu z}\\ \times \int_{0}^{2\pi }\int_{0}^{\pi }\int_{0}^{\beta }drd\theta d\varphi \;r^{2}\sin\theta \;r^{L}Y_{L,M}(\theta ,\varphi )\exp(-\alpha r^{2})\sum_{\ell =0}^{\infty }i_{\ell }(2\alpha Rr)\sum_{m=-\ell }^{\ell }Y_{\ell ,m}{}^{*}(\theta ,\varphi )Y_{\ell ,m}^{}(\theta_{R},\varphi_{R}){\left(r\sin\theta \cos\varphi \right)}^{\lambda }{\left(r\sin\theta \sin\varphi \right)}^{\mu }{\left(r\cos\theta \right)}^{\nu } \end{array}$$

Further rearrangement yields the following master equation, eq. (18),

(18) $$\begin{array}{l} Q_{LM}(\beta )=\;\;\sqrt{\frac{{\left(4\pi \right)}^{3}}{2L+1}}\;\;K\exp(-\alpha R_{}^{2})\sum_{\lambda =0}^{}\sum_{\mu =0}^{}\sum_{\nu =0}^{}f_{\lambda x}f_{\mu y}f_{\nu z}\\ \times \sum_{\ell =0}^{\infty }\int_{0}^{\beta }dr\;r^{\lambda +\mu +\nu +L+2}\exp(-\alpha r^{2})\;i_{\ell }(2\alpha Rr)\sum_{m=-\ell }^{\ell }Y_{\ell ,m}^{}(\theta_{R},\varphi_{R})\int_{0}^{2\pi }\int_{0}^{\pi }d\theta d\varphi \;Y_{\ell ,m}{}^{*}(\theta ,\varphi )Y_{L,M}(\theta ,\varphi )\sin^{\lambda +\mu +1}\theta \cos^{\nu }\theta \sin^{\mu }\varphi \cos^{\lambda }\varphi \end{array}$$

In the special case where *λ* = *ν* = *μ* = 0, the angular integration in eq. (18) has been prepared for the use of the orthonormalization relationship announced above,

(19) $$\int_{0}^{\pi }\int_{0}^{2\pi }\;d\theta \;d\varphi \sin\theta \;Y_{\ell^{\prime },m^{\prime }}^{*}(\theta ,\varphi )\;Y_{\ell ,m}(\theta ,\varphi )=\;\;\delta_{\ell \ell^{\prime }}\delta_{mm^{\prime }}$$

such that eq. (18) becomes

(20) $$\begin{array}{l} Q_{LM}(\beta )=\;\;\sqrt{\frac{{\left(4\pi \right)}^{3}}{2L+1}}\;\;K\exp(-\alpha R_{}^{2})\sum_{\ell =0}^{\infty }\int_{0}^{\beta }dr\;r^{L+2}\exp(-\alpha r^{2})\;i_{\ell }(2\alpha Rr)\sum_{m=-\ell }^{\ell }Y_{\ell ,m}^{}(\theta_{R},\varphi_{R})\;\delta_{\ell L}\delta_{mM}\\ \;\;\;=\sqrt{\frac{{\left(4\pi \right)}^{3}}{2L+1}}\;\;K\exp(-\alpha R_{}^{2})\;Y_{L,M}^{}(\theta_{R},\varphi_{R})\int_{0}^{\beta }dr\;r^{L+2}\exp(-\alpha r^{2})\;i_{L}(2\alpha Rr)\; \end{array}$$

This pleasing result shows that there is no need to evaluate any angular integrals in the case of only s‐like Gaussian primitives contributing to *Q_LM_*(*β*). Second, the multipole moment *Q_LM_*(*β*) is simply proportional to the value of *Y_L,M_*(*θ*_R_, *ϕ*_R_). Note that this multipole moment *Q_LM_*(*β*) can be a complex number because *Y_L,M_* can be complex and the other factors in eq. (20) are real. Third, the contribution of a given Gaussian primitive drops off quickly with the distance of its center from the origin **o**. The radial integral in eq. (20), at any order *L*, can be reduced to a linear combination of the basic integral of an exponential with quadratic argument. An efficient implementation to any order *L* is possible through a recursion relation for modified spherical Bessel functions.

We now return to the master equation, eq. (18), which presents a radial and a double angular integral to be solved. The radial integral is tackled first, and can be written as

(21) $$I_{\text{rad}}(\ell ,S\;;\alpha ,\beta ,p)=\int_{0}^{\beta }dr\;r^{\lambda +\mu +\nu +L+2}\exp(-\alpha r^{2})\;i_{\ell }(2\alpha Rr)=\int_{0}^{\beta }dr\;r^{S}\exp(-\alpha r^{2})\;i_{\ell }(pr)$$

where *p = 2αR* and *S* = *λ* + *μ* + *υ* + *L* + 2 simplify the notation of the integral. This integral does not appear to have been solved based on ready inspection of published solved integrals. Therefore, the integral had to be creatively reduced to known integrals. The explicit derivation and validation has been siphoned off to Part D in the Supporting Information, but we quote the final result here,

(22) $$I_{f}^{\pm }(s)=\int_{0}^{\beta }dr\;r^{s}\exp(-\alpha r^{2}\pm pr)=\exp(\frac{p^{2}}{4\alpha })\sum_{k=0}^{s}\left(\begin{array}{c} s\\ k \end{array}\right)\;\frac{{\left(\pm \frac{p}{2\alpha }\right)}^{s-k}}{2\alpha^{\left(\frac{k+1}{2}\right)}}\left[\Gamma \left(\frac{k+1}{2},\alpha {\left(\mp p/2\alpha \right)}^{2}\right)-\Gamma \left(\frac{k+1}{2},\alpha {\left[\beta \mp (p/2\alpha )\right]}^{2}\right)\right]$$

where Γ(*a*,*x*) is the upper incomplete gamma function (or of the second kind). Part D of the Supporting Information shows how this solved integral cascades up, via a recursive relation, to the solution of $I_{\text{rad}}(\ell ,S\;;\alpha ,\beta ,p)$. Note that if *a* is an integer then Γ(*a*,*x*) returns essentially an exponential, while if *a* is a half‐integer then the error function emerges. Also note that the ∓ sign must be explicitly preserved when taking the square, because if a square root is taken of this square product it is important that the original sign is preserved.

The second sizeable task in solving the master integral in eq. (18) focuses on $I_{\text{ang}}(L,M,l,m,\lambda ,\mu ,\nu )$, the angular part counterpart of the radial integral $I_{\text{rad}}(\ell ,S\;;\alpha ,\beta ,p)$. To shorten this article even more, the explicit derivation and validation has been siphoned off to Part E in the Supporting Information. This derivation solves the angular integral analytically, splitting it into an integral in *θ* and one in *φ*, that is,

(23) $$\begin{array}{l} I_{\text{ang}}(L,M,l,m,\lambda ,\mu ,\nu )=\int_{0}^{2\pi }\int_{0}^{\pi }d\theta d\varphi \;Y_{\ell ,m}{}^{*}(\theta ,\varphi )Y_{L,M}(\theta ,\varphi )\sin^{\lambda +\mu +1}\theta \;\cos^{\nu }\theta \;\sin^{\mu }\varphi \;\cos^{\lambda }\varphi \\ \;\;={\left[\frac{\left(2\ell +1\right)}{4\pi }\frac{(\ell -m)!}{(\ell +m)!}\right]}^{1/2}{\left[\frac{\left(2L+1\right)}{4\pi }\frac{(L-M)!}{(L+M)!}\right]}^{1/2}\int_{0}^{\pi }d\theta \;P_{\ell }^{m}(\cos\theta )P_{L}^{M}(\cos\theta )\sin^{\lambda +\mu +1}\theta \;\cos^{\nu }\theta \int_{0}^{2\pi }d\varphi \;e^{i(M-m)\varphi }\sin^{\mu }\varphi \;\cos^{\lambda }\varphi \\ \;\;={\left[\frac{\left(2\ell +1\right)}{4\pi }\frac{(\ell -m)!}{(\ell +m)!}\right]}^{1/2}{\left[\frac{\left(2L+1\right)}{4\pi }\frac{(L-M)!}{(L+M)!}\right]}^{1/2}I_{\theta }(L,M,l,m,\lambda ,\mu ,\nu )I_{\varphi }(M,m,\lambda ,\mu ) \end{array}$$

The result for the integral in φ is

(24) $$I_{\varphi }(M,m,\lambda ,\mu )=\frac{\;\pi }{2^{\lambda +\mu -1}}{\left(-i\right)}^{\mu }\sum_{k=0}^{\mu }{\left(-1\right)}^{\mu -k}\left(\begin{array}{c} \mu \\ k \end{array}\right)\;\left(\begin{array}{c} \lambda \\ \frac{1}{2}(\lambda +\mu +m-M)-k \end{array}\right)\;\text{if}\;\lambda +\mu +\left|m-M\right|\;\text{is}\;\text{even}$$

It is very important to realize that *I_φ_* vanishes if *m* > *λ* + *μ* + *M*.

In the calculation of *θ*, it proved crucial to use a finite expansion of the associated Legendre polynomials in terms of cosines and sines, to obtain a closed and more importantly, universal expression, which is

(25) $$\begin{array}{l} I_{\theta }(L,M,l,m,\lambda ,\mu ,\nu )={\left(-\frac{1}{2}\right)}^{m+M}(\ell +m)!(L+M)!\sum_{j=0}^{\left[\frac{\ell -m}{2}\right]}\sum_{k=0}^{\left[\frac{L-M}{2}\right]}{\left(-\frac{1}{4}\right)}^{j+k}\frac{1}{(\ell -m-2j)!(m+j)!j!(L-M-2k)!(M+k)!k!}\times \\ \;\;\;\;\;\;\left[1+{\left(-1\right)}^{a}\right]\frac{\Gamma \left(\frac{a+1}{2}\right)\Gamma \left(\frac{b+1}{2}\right)}{2\Gamma \left(\frac{a+b+2}{2}\right)}\;\;\text{where}\;a=l-m-2j+L-M-2k+v\;\text{and}\;b=m+2j+M+2k+\lambda +\mu +1 \end{array}$$

In summary, by substituting eqs. (24) and (25) back into eq. (23), the integral in the master equation eq. (18) has been solved. Thus, we obtained a general formula for an arbitrary spherical harmonic multipole moment generated by the electron density, within a sphere with radius *β*, and constructed from Gaussian of arbitrary angular momentum.

The next derivation focuses on the kinetic energy, starting with one type of kinetic energy density, usually denoted *G*(**r**) but here written as *E*_G_(**r**) to avoid confusion with the Gaussian primitive function. Because *E*_G_(**r**) contains only first derivatives the expressions are less complex than for another type of kinetic energy density called *K*(**r**), now denoted *E*_K_(**r**) for consistency. The latter contains second derivatives and is discussed later. The contribution of the *β* sphere to the integrated kinetic energy, denoted *E*_G_(*β*), is defined as follows:

(26) $$E_{G}(\beta )=\int_{\beta \;\;\text{sphere}}dr\;E_{G}(r)=\frac{1}{2}\sum_{p=1}^{n_{\text{MO}}}n_{p}\int_{\beta \;\;\text{sphere}}dr\nabla \psi_{p}^{}•\nabla \psi_{p}^{}$$

where all symbols in common with eq. (3) mean the same. The derivation leading to a solution for eq. (26) is given in Part F of the Supporting Information. Thus, the equivalent of eq. (11), which formulates the key integral for *Q_LM,jk_*_,_ then becomes

(27) $$E_{G,jk}(\beta )=\;K_{jk}\sum_{r=1}^{12}F_{jk,r}\sum_{\lambda =0}^{}\sum_{\mu =0}^{}\sum_{\nu =0}^{}f_{\lambda x,r}f_{\mu y,r}f_{\nu z,r}\int_{\beta }^{}dr\;\;\exp(-\alpha_{jk}{|r-R_{jk}|}^{2})x^{\lambda }y^{\mu }z^{\nu }$$

This equation concludes the proof that the G‐type kinetic energy can be calculated by closed expressions because the integral in eq. (27) is a special case (i.e., *L* = *M* = 0 in *R_LM_*(**r**)) of the integral in eq. (11). Hence, the treatment of the integral in eq. (27) is identical of that in eq. (11). Finally, it should be pointed out that the gradient operator never appeared expressed in spherical polar coordinates. Indeed, its differentiation was carried out at Cartesian level, prior to the treatment of the complex integrals in spherical polar coordinates. The same strategy is followed for the second type of kinetic energy denoted *E*_K_(**r**).

The next derivation (or rather outline thereof) focuses on *E*_K_(**r**), where the contribution of the *β* sphere to the integrated kinetic energy, denoted *E*_K_(*β*), is defined as follows:

(28) $$E_{K}(\beta )=\int_{\beta \;\;}dr\;E_{K}(r)=-\frac{1}{2}\sum_{p=1}^{n_{\text{MO}}}n_{p}\int_{\beta \;\;}dr\psi_{p}^{}\nabla^{2}\psi_{p}^{}$$

One can prove that the Laplacian operator operating on a Gaussian primitive again results in a linear combination of Gaussian primitives:

(29) $$\begin{array}{l} \nabla^{2}G_{j}(r-R_{j};\alpha_{j},{\bar{l}}_{j},{\bar{m}}_{j},{\bar{n}}_{j})=-2\alpha_{j}\left[2({\bar{l}}_{j}+{\bar{m}}_{j}+{\bar{n}}_{j})+3\right]G_{j}(r-R_{j};\alpha_{j},{\bar{l}}_{j},{\bar{m}}_{j},{\bar{n}}_{j})\\ \;\;+4\alpha_{j}^{2}\left[G_{j}(r-R_{j};\alpha_{j},{\bar{l}}_{j}+2,{\bar{m}}_{j},{\bar{n}}_{j})+G_{j}(r-R_{j};\alpha_{j},{\bar{l}}_{j},{\bar{m}}_{j}+2,{\bar{n}}_{j})+G_{j}(r-R_{j};\alpha_{j},{\bar{l}}_{j},{\bar{m}}_{j},{\bar{n}}_{j}+2)\right]\\ \;\;+\left[{\bar{l}}_{j}({\bar{l}}_{j}-1)G_{j}(r-R_{j};\alpha_{j},{\bar{l}}_{j}-2,{\bar{m}}_{j},{\bar{n}}_{j})+{\bar{m}}_{j}({\bar{m}}_{j}-1)G_{j}(r-R_{j};\alpha_{j},{\bar{l}}_{j},{\bar{m}}_{j}-2,{\bar{n}}_{j})+{\bar{n}}_{j}({\bar{n}}_{j}-1)G_{j}(r-R_{j};\alpha_{j},{\bar{l}}_{j},{\bar{m}}_{j},{\bar{n}}_{j}-2)\right] \end{array}$$

This equation allows one to write the equivalent of eq. (F4) of the Supporting Information:

(30) $$\begin{array}{l} E_{K}(\beta )=\;-\frac{1}{2}\sum_{p=1}^{n_{\text{MO}}}n_{p}\int_{\beta \;\;}dr\left(\sum_{j=1}^{n_{G}}c_{jp}G_{j}(r-R_{j};\alpha_{j},{\bar{l}}_{j},{\bar{m}}_{j},{\bar{n}}_{j})\right)\left(\sum_{s=1}^{3}\frac{\partial^{2}}{\partial^{2}q_{s}}\left(\sum_{k=1}^{n_{G}}c_{kp}G_{k}(r-R_{k};\alpha_{k},{\bar{l}}_{k},{\bar{m}}_{k},{\bar{n}}_{k})\right)\right)\\ =-\frac{1}{2}\sum_{p=1}^{n_{\text{MO}}}n_{p}\sum_{j=1}^{n_{G}}\sum_{k=1}^{n_{G}}c_{kp}c_{jp}\int_{\beta \;\;}dr\sum_{r=1}^{7}F_{jk,r}^{K}G_{jk}(r-R_{jk};\alpha_{jk},{\bar{l}}_{jk,r},{\bar{m}}_{jk,r},{\bar{n}}_{jk,r})\\ =-\frac{1}{2}\sum_{p=1}^{n_{\text{MO}}}n_{p}\sum_{j=1}^{n_{G}}\sum_{k=1}^{n_{G}}c_{kp}c_{jp}E_{K,jk}(\beta ) \end{array}$$

where the explicit product between *G_j_* and $\nabla^{2}G_{k}$ is now not written out but seen to give rise to a 7‐term sum with its own pre‐factors *F*, and pattern of powers (*l*, *m*, *n*). As for *E*_G_, we are now re‐assured that we make contact with the previously established machinery of solving the arising integrals. This completes the treatment of kinetic energy. Note that the *β*‐sphere contribution of the Laplacian of the electron density can be trivially obtained from the two kinetic energies because

(31) $$L(\beta )=\;-\frac{1}{4}\int_{\beta \;\;}dr\;\nabla^{2}\rho (r)=E_{K}(\beta )-E_{G}(\beta )\;$$

To complete the treatment of IQA energies by their *β*‐sphere contributions, the final section focuses on the potential energy, both within the same topological atom (intra) and between two different topological atoms (inter). An efficient strategy to calculate potential energies builds on two insights: (i) the calculation of the electrostatic potential (generated by a given electron density) as a useful intermediate physical quantity, and (ii) the exploitation of the exact convergence of the multipole expansion (due to the finite volume of the *β*‐sphere).

**Figure 2:**
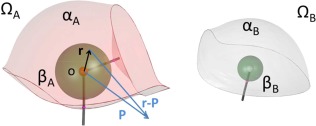
Electrostatic potential and potential energies within and between two topological atoms Ω_A_ and Ω_B_. Note that for convenience the global origin coincides with the nuclear position of Ω_A_. [Color figure can be viewed at wileyonlinelibrary.com]

Figure 2 sets the scene, introducing symbols that will appear in the equations below. A topological atom, denoted Ω, consists of two regions: the *β*‐sphere and the remaining region outside the *β*‐sphere, which we call “*α*.” All integration in the *α*‐region is done by numerical quadrature, typically by a Gauss‐Legendre for the radial part and a Lebedev grid for the angular part. The integration of the complex shape of the *α*‐region could be possible analytically as well, by introducing so‐called natural coordinates in eq. (18), but actually achieving this is perhaps more of a retirement project. The point **P** marks a possible position where one wants to know the electrostatic potential generated by the electron density in *β*_A_.

The electrostatic potential at a given point **P,** generated by the total charge density inside the *β* sphere, is defined by

(32) $$V_{\text{tot}}\left(P\right)=\int_{\beta }dr\frac{\rho_{\text{tot}}\left(r\right)}{\;\left|\;r-P\right|}=\frac{Z_{\beta }}{\left|\;r\right|}-\int_{\beta }dr\frac{\rho \left(r\right)}{\;\left|\;r-P\right|}=V_{\text{nuc}}+V_{\text{elec}}$$

where *ρ*_tot_(**r**) is the sum of the electronic and nuclear charge density, described by the position vector **r,** *ρ*(**r**) the purely electronic density featuring throughout this article [starting with eq. (1)], and Z_*β*_ is the charge of the nucleus inside the *β* sphere. We take advantage of the following well known expansion

(33) $$\frac{1}{\left|r-P\;\right|}=\sum_{L=0}^{\infty }\frac{r_{<}^{L}}{r_{>}^{L+1}}\left(\frac{4\pi }{2L+1}\right)\sum_{M=-L}^{L}{\left(-1\right)}^{M}Y_{L,-M}(\theta_{P},\varphi_{P})Y_{L,M}(\theta ,\varphi )\;\;\;\;\;\;\;\;$$

In this expression, $r_{<}$ is the smaller and $r_{>}$ is the larger value and the common choice is to assign *r* = |**r**| to $r_{<}$, and to assign *P* = |**P**| (where both vectors **r** and **P** refer to the same global origin) to $r_{>}$. This expansion will then converge, provided *r* < *P*, which means that the point **P** must lie outside the *β* sphere. This is perfectly possible because the electron density is confined to the *β* sphere. In other words, formal convergence is only possible if the point (**P**) at which the potential is evaluated lies further from the nucleus than any element of electron density within the *β*‐sphere. Substituting eq. (33) into eq. (32) leads to

(34) $$V_{\text{elec}}\left(P\right)=-\int_{\beta }dr\frac{\rho \left(r\right)}{\;\left|\;r-P\right|}=-\sum_{L=0}^{\infty }{\left(\frac{4\pi }{2L+1}\right)}^{1/2}\frac{1}{P_{}^{L+1}}\sum_{M=-L}^{L}{\left(-1\right)}^{M}Y_{L,-M}(\theta_{P},\varphi_{P})\underset{Q_{LM}(\beta )}{\underset{︸}{\int_{\beta }dr\rho \left(r\right)R_{L,M}(\theta ,\varphi )}}$$

which proves that the electrostatic potential can be calculated directly from the previously computed multipole moments *Q_LM_*(*β*), without introducing new integrals. The calculation of the potential energy between two topological atoms benefits from this straightforwardly calculated electrostatic potential because this energy can be written as follows:

(35) $$E_{\text{AB}}^{\text{pot}}=\int_{\Omega_{A}}dr_{A}\int_{\Omega_{B}}dr_{B}\;\frac{\rho_{\text{tot}}^{}(r_{A})\;\rho_{\text{tot}}^{}(r_{B})}{r_{\text{AB}}}=\int_{\Omega_{A}}dr_{A}\;\rho_{\text{tot}}^{}(r_{A})V_{B}(r_{A})=Z_{A}V_{B}(R_{A})-\int_{\Omega_{A}}dr_{A}\;\rho (r_{A})V_{B}(r_{A})$$

where

(36) $$V_{B}(r_{A})=\int_{\Omega_{B}}dr_{B}\;\frac{\rho_{\text{tot}}^{}(r_{B})\;}{r_{\text{AB}}}$$

is the electrostatic potential generated by atom B in atom A.

It is important to fully exploit the fact that the electrostatic potential generated by the *β* sphere is analytically computed in any point outside it. Because each atom is a union of *β* sphere and an *α* region, or Ω = *α* U *β*, there are four possible interactions, between two different atoms (A ≠ B): *β*_A_ – *β*_B_, *β*_A_ – *α*_B_, *α*_A_ – *β*_B_, and *α*_A_ – *α*_B_. This division can be summarized as follows:

(37) $$\begin{array}{c} E_{\text{AB}}^{\text{pot}}=\;\;\underset{\text{analytical}}{\underset{︸}{\int_{\beta_{A}}dr_{1}\int_{\beta_{B}}dr_{2}\;\;\frac{\rho_{\text{tot}}^{}(r_{1})\;\rho_{\text{tot}}^{}(r_{2})}{r_{12}}}}\;\;+\underset{\text{numerical}-\text{analytical}}{\underset{︸}{\int_{\beta_{A}}dr_{1}\int_{\alpha_{B}}dr_{2}\;\;\frac{\rho_{\text{tot}}^{}(r_{1})\;\rho_{\text{tot}}^{}(r_{2})}{r_{12}}}}\;\;\;\\ +\underset{\text{numerical}-\text{analytical}}{\underset{︸}{\int_{\alpha_{A}}dr_{1}\int_{\beta_{B}}dr_{2}\;\;\frac{\rho_{\text{tot}}^{}(r_{1})\;\rho_{\text{tot}}^{}(r_{2})}{r_{12}}}}\;+\underset{\text{numerical}}{\underset{︸}{\int_{\alpha_{A}}dr_{1}\int_{\alpha_{B}}dr_{2}\;\;\frac{\rho_{\text{tot}}^{}(r_{1})\;\rho_{\text{tot}}^{}(r_{2})}{r_{12}}\;}}\\ =\sum_{L_{A}L_{B}M_{A}M_{B}}T_{L_{A}L_{B}M_{A}M_{B}}(R_{\text{AB}}^{})\;Q_{L_{A}M_{A}}^{}(\beta_{A})\;Q_{L_{B}M_{B}}^{}(\beta_{B})+\int_{\alpha_{B}}dr_{2}\;\rho_{\text{tot}}^{}(r_{2})V_{\beta_{A}}(r_{2})\\ +\int_{\alpha_{A}}dr_{1}\;\rho_{\text{tot}}^{}(r_{1})V_{\beta_{B}}(r_{1})+\int_{\alpha_{A}}dr_{1}\int_{\alpha_{B}}dr_{2}\;\;\frac{\rho_{\text{tot}}^{}(r_{1})\;\rho_{\text{tot}}^{}(r_{2})}{r_{12}}\; \end{array}$$

where $T_{L_{A}M_{A}L_{B}M_{B}}$ is a purely geometric interaction tensor and **R**_AB_ is the internuclear vector. In the second and third term, the quadrature grid over the respective *α*‐regions of atoms *B* and *A* will ask for values of the analytically evaluated electrostatic potential in each of its points. The fourth term is purely numerical and hence does not benefit from the work of the current article. There remains only one case to be mentioned, which is that of A = B, because it causes its own computational regime, but only for *β*_A_ – *β*_A_. Indeed, the interactions *β*_A_ – *α*_A_ and *α*_A_ – *α*_A_ can be calculated without extra knowledge. For the *β*_A_ – *β*_A_ case, one option is to follow the method “inverse multipole moments,”^54^ another is to introduce a Fourier transform that is used in the evaluation of nuclear‐electron attraction integrals.

## Conclusions

We present a detailed derivation of a fully analytical 3D integration over the volume bounded by the *β* sphere inside a topological atom. The formulae are sufficiently general such that a Gaussian primitive of arbitrary angular momentum can contribute to the electron density within the *β* sphere. We obtained a general formula for an arbitrary spherical harmonic multipole moment, the kinetic energies and the potential energy between atoms. We showed that closed expressions can be obtained involving the exponential, error function, and gamma functions. The implementation of the results of the derivations given here will follow in a subsequent publication. This implementation will make a choice of quadrature grid superfluous, but it is not yet clear how the current formulae will perform computationally, compared with quadrature schemes.

## Supporting information

Supporting Information
