# Supplementary material for: A fully analytical integration of properties over the 3D volume of the β sphere in topological atoms
Source: J Comput Chem. 2018 Jan 10;39(10):604–13. doi: 10.1002/jcc.25158 (PMC5838411; doi:10.1002/jcc.25158)
Supplement: Supplementary file 1 — Supporting Information [file JCC-39-604-s001.pdf]

Supporting Information

A Fully Analytical Integration of Properties over the 3D Volume of the β Sphere in Topological Atoms

Paul Popelier

Manchester Institute of Biotechnology (MIB)

131 Princess Street, Univ. of Manchester, Manchester M1 7DN, Great Britain

and School of Chemistry, Univ. of Manchester, Oxford Road, Manchester M13 9PL, Great Britain

Part A: Regular spherical (solid) harmonics

In physics the spherical harmonic function $Y_{lm}(\theta, \varphi)$ is defined as in eq A1,

$$Y_{\ell,m}(\theta,\varphi) = \left[\frac{(2\ell+1)}{4\pi} \frac{(\ell-m)!}{(\ell+m)!} \right]^{1/2} P_\ell^m(\cos\theta) e^{im\varphi} \qquad \text{(A1)}$$

where P_l^m is an *associated Legendre function*, and θ is the polar angle ($0 \leq \theta \leq \pi$) while φ is the azimuth ($0 \leq \varphi \leq 2\pi$). We note that P_{lm} is also used where $P_{lm}=(-1)^m P_l^m$. It should also be realised that the so-called surface harmonics Y_l^m are not the same as $Y_{l,m}$ (see 14.30.2 in the NIST handbook[1]). However, the Wikipedia entry on Spherical Harmonics ignores this distinction and writes Y_l^m instead of $Y_{l,m}$ in eq A1. We follow the NIST handbook. In the physicist's convention, the North Pole corresponds to θ = 0 and the Equator to θ = $\pi/2$.

Is eq A1 consistent with the explicit expressions given for spherical harmonics given under the Wikipedia entries "Table of spherical harmonics" and "Associated Legendre polynomials"? It is sufficient to investigate this question for ℓ = 1 and m = ±1.

$$Y_{1,-1}(\theta,\varphi) = \left[\frac{(2+1)}{4\pi} \frac{(1-(-1))!}{(1+(-1))!} \right]^{1/2} P_1^{-1}(\cos\theta) e^{-i\varphi} = \sqrt{\frac{3}{2\pi}} (\frac{1}{2}\sin\theta) e^{-i\varphi} = \frac{1}{2}\sqrt{\frac{3}{2\pi}} \frac{(x-iy)}{r} \qquad \text{(A2)}$$

$$Y_{1,1}(\theta,\varphi) = \left[\frac{(2+1)}{4\pi} \frac{(1-1)!}{(1+1)!} \right]^{1/2} P_1^{1}(\cos\theta) e^{i\varphi} = \sqrt{\frac{3}{8\pi}} (-\sin\theta) e^{i\varphi} = -\frac{1}{2}\sqrt{\frac{3}{2\pi}} \frac{(x+iy)}{r} \qquad \text{(A3)}$$

Equations A2 and A3 answer the aforementioned question affirmatively. This means that a definition of $Y_{l,m}$ (in eq A1), which involves |m| instead of *m*, is not compatible with eq A3. With |m| the expression for $Y_{1,-1}$ would end up smaller by a factor 2. Note that the Condon-Shortley phase has been employed in the above. This factor never appears in the geodesy and magnetics community but serves the quantum mechanics community by simplifying some operations. Although there is no need to know this factor explicitly, it is useful to know that it is $(-1)^m$ if m > 0 and 1 otherwise.

The Wikipedia entry "Solid Harmonics" defines regular solid (or spherical) harmonics as

$$R_{\ell,m}(\mathbf{r}) = R_{\ell,m}(r,\theta,\varphi) = r^\ell C_{\ell,m}(\theta,\varphi) = r^\ell \left[\frac{4\pi}{(2\ell+1)} \right]^{1/2} Y_{\ell,m}(\theta,\varphi) \qquad \text{(A4)}$$

These regular spherical harmonics define the spherical tensor QCT multipole moments. The simplest regular spherical harmonic is that associated with the monopole moment is,

$$R_{00}(\mathbf{r}) = 1 \qquad \text{(A5)}$$

This is a particularly simple expression, due to the prefactor introduced in eq A4, because working with the original spherical harmonic $Y_{l,m}(\theta, \varphi)$ would give $Y_{0,0}(\theta,\varphi) = \sqrt{1/4\pi}$.

Using the Wikipedia entries mentioned above, the regular spherical harmonics associated with the dipole moments are given, for now, by eqs A6, A7 and A8,

$$R_{1,0}(\mathbf{r}) = r\left(\frac{4\pi}{3}\right)^{1/2}\left[\frac{1}{2}\sqrt{\frac{3}{\pi}}\cos\theta\right] = r\cos\theta = z \qquad \text{(A6)}$$

$$R_{1,-1}(\mathbf{r}) = r\sqrt{\frac{4\pi}{3}}\left[\sqrt{\frac{3}{2\pi}}(\frac{1}{2}\sin\theta)e^{-i\varphi}\right] = \sqrt{\frac{1}{2}}r\sin\theta\, e^{-i\varphi} = \sqrt{\frac{1}{2}}r\sin\theta(\cos\varphi - i\sin\varphi) = \sqrt{\frac{1}{2}}(x - iy) \qquad \text{(A7)}$$

$$R_{1,1}(\mathbf{r}) = r\sqrt{\frac{4\pi}{3}}\left[\sqrt{\frac{3}{8\pi}}(-\sin\theta)e^{i\varphi}\right] = -\sqrt{\frac{1}{2}}r\sin\theta\, e^{i\varphi} = -\sqrt{\frac{1}{2}}r\sin\theta(\cos\varphi + i\sin\varphi) = -\sqrt{\frac{1}{2}}(x + iy) \qquad \text{(A8)}$$

In order to eliminate complex numbers the following real-function equivalents of $R_{\ell,m}(\mathbf{r})$ are introduced, where m > 0, and *c* and *s* refer to cosine and sine, respectively,

$$R_{\ell mc}(\mathbf{r}) = \sqrt{\frac{1}{2}}[R_{\ell,-m}(\mathbf{r}) + (-1)^m R_{\ell,m}(\mathbf{r})] \qquad \text{(A9)}$$

$$R_{\ell ms}(\mathbf{r}) = i\sqrt{\frac{1}{2}}[R_{\ell,-m}(\mathbf{r}) - (-1)^m R_{\ell,m}(\mathbf{r})] \qquad \text{(A10)}$$

Eqs A9 and A10 cannot be applied to $R_{\ell 0}(\mathbf{r})$ because m=0 is incompatible with the requirement that *m* must be strictly larger than zero. However, for m=1 we obtain

$$R_{11c}(\mathbf{r}) = \sqrt{\frac{1}{2}}[R_{1,-1}(\mathbf{r}) - R_{1,1}(\mathbf{r})] = \frac{1}{2}[(x - iy) + (x + iy)] = x \qquad \text{(A11)}$$

$$R_{11s}(\mathbf{r}) = i\sqrt{\frac{1}{2}}[R_{1,-1}(\mathbf{r}) - R_{1,1}(\mathbf{r})] = \frac{i}{2}[(x - iy) - (x - iy)] = \frac{i}{2}(-2iy) = y \qquad \text{(A12)}$$

Part B: Some explicit formulae for the angular part of a product Gaussian primitive

Applying eq. (9) in the main text for the power λ=r+s=2, where the pair (r, s) can obtain three possible values, i.e. (r, s)=(0,2) ; (r, s)=(1,1) or (r, s)=(2,0), yields

$$\begin{aligned} f_{2x} &= \binom{\overline{\ell}_j}{0}\binom{\overline{\ell}_k}{2}(-X_j)^{\overline{\ell}_j}(-X_k)^{\overline{\ell}_k-2} + \binom{\overline{\ell}_j}{1}\binom{\overline{\ell}_k}{1}(-X_j)^{\overline{\ell}_j-1}(-X_k)^{\overline{\ell}_k-1} + \binom{\overline{\ell}_j}{2}\binom{\overline{\ell}_k}{0}(-X_j)^{\overline{\ell}_j-2}(-X_k)^{\overline{\ell}_k} \\ &= \frac{1}{2}\overline{\ell}_k(\overline{\ell}_k-1)(-X_j)^{\overline{\ell}_j}(-X_k)^{\overline{\ell}_k-2} + \overline{\ell}_j\overline{\ell}_k(-X_j)^{\overline{\ell}_j-1}(-X_k)^{\overline{\ell}_k-1} + \frac{1}{2}\overline{\ell}_j(\overline{\ell}_j-1)(-X_j)^{\overline{\ell}_j-2}(-X_k)^{\overline{\ell}_k} \end{aligned} \quad \text{(B1)}$$

Applying eq. (9) for the power μ=r+s=3, now covering four possible values of the pair (r, s), i.e. (r, s)=(0,3) ; (r, s)=(1,2) ; (r, s)=(2,1) or (r, s)=(3,0), yields

$$\begin{aligned} f_{3x} &= \binom{\overline{\ell}_j}{0}\binom{\overline{\ell}_k}{3}(-X_j)^{\overline{\ell}_j}(-X_k)^{\overline{\ell}_k-3} + \binom{\overline{\ell}_j}{1}\binom{\overline{\ell}_k}{2}(-X_j)^{\overline{\ell}_j-1}(-X_k)^{\overline{\ell}_k-2} \\ &\quad + \binom{\overline{\ell}_j}{2}\binom{\overline{\ell}_k}{1}(-X_j)^{\overline{\ell}_j-2}(-X_k)^{\overline{\ell}_k-1} + \binom{\overline{\ell}_j}{3}\binom{\overline{\ell}_k}{0}(-X_j)^{\overline{\ell}_j-3}(-X_k)^{\overline{\ell}_k} \\ &= \frac{1}{6}\overline{\ell}_k(\overline{\ell}_k-1)(\overline{\ell}_k-2)(-X_j)^{\overline{\ell}_j}(-X_k)^{\overline{\ell}_k-3} + \frac{1}{2}\overline{\ell}_j\overline{\ell}_k(\overline{\ell}_k-1)(-X_j)^{\overline{\ell}_j-1}(-X_k)^{\overline{\ell}_k-2} \\ &\quad + \frac{1}{2}\overline{\ell}_j(\overline{\ell}_j-1)\overline{\ell}_k(-X_j)^{\overline{\ell}_j-2}(-X_k)^{\overline{\ell}_k-1} + \frac{1}{6}\overline{\ell}_j(\overline{\ell}_j-1)(\overline{\ell}_j-2)(-X_j)^{\overline{\ell}_j-3}(-X_k)^{\overline{\ell}_k} \end{aligned} \quad \text{(B2)}$$

Applying eq. (11) in the main text is helpful in the context of the case shown in Figure 1 of the main text, where two different p-type Gaussian primitives (p_y and p_z) contribute to $Q_{LM,\,jk}$. We then obtain

$$Q_{LM,jk}(\beta) = K_{jk}\sum_{\mu=0}^{1}\sum_{\nu=0}^{1} f_{\mu y}(Y_j,Y_k) f_{\nu z}(Z_j,Z_k)\int_{\beta} d\mathbf{r}\, R_{LM}(\mathbf{r})\exp(-\alpha\left|\mathbf{r}-\mathbf{R}_{jk}\right|^2) y^{\mu} z^{\nu} \quad \text{(B3)}$$

By direct calculation we obtain eq B4:

$$\begin{aligned} Q_{LM,jk}(\beta) &= K_{jk}\int_{\beta} d\mathbf{r}\, R_{LM}(\mathbf{r})\exp(-\alpha\left|\mathbf{r}-\mathbf{R}_{jk}\right|^2)(y-Y_j)(z-Z_k) \\ &= K_{jk}Y_jZ_k\int_{\beta} d\mathbf{r}\, R_{LM}(\mathbf{r})\exp(-\alpha\left|\mathbf{r}-\mathbf{R}_{jk}\right|^2) - K_{jk}\int_{\beta} d\mathbf{r}\, R_{LM}(\mathbf{r})\exp(-\alpha\left|\mathbf{r}-\mathbf{R}_{jk}\right|^2)(yZ_k+Y_jz) \\ &\quad + K_{jk}\int_{\beta} d\mathbf{r}\, R_{LM}(\mathbf{r})\exp(-\alpha\left|\mathbf{r}-\mathbf{R}_{jk}\right|^2)yz \end{aligned} \quad \text{(B4)}$$

It is easy to prove that eq B3 and B4 are identical, using eq. (11) and the fact that $f_{0x}=1$ (while appearing just as a single factor in the product of eq. (11)), and the fact that $(\overline{\ell}_j,\overline{m}_j,\overline{n}_j)=(0,1,0)$ (for p_y) and $(\overline{\ell}_k,\overline{m}_k,\overline{n}_k)=(0,0,1)$ (for p_z). The equivalent of eq. (10) of the main text is then

$$\begin{aligned} f_{0y}(Y_j,Y_k) &= (-Y_j)^{\overline{m}_j}(-Y_k)^{\overline{m}_k} \\ f_{1y}(Y_j,Y_k) &= \overline{m}_k(-Y_j)^{\overline{m}_j}(-Y_k)^{\overline{m}_k-1} + \overline{m}_j(-Y_j)^{\overline{m}_j-1}(-Y_k)^{\overline{m}_k} \end{aligned} \quad \text{(B5)}$$

As a result, we obtain that $f_{0y}= -Y_j$, $f_{1y} = 1$. Aware of the equivalent of eq B5 for the z-component, we obtain $f_{0z} = -Z_k$ and $f_{1z} = 1$.

Part C: Derivation of the key equation eq 12 of the main text

The reason that eq 12 of the main text is a key equation is that this equation enables to shift a Gaussian primitive from one centre to another. Equation 12 has been given before (as eq 7 in reference[2]). However, here we independently derive it, starting from the better known plane-wave expansion, also known as the Rayleigh equation. This expansion is textbook material and commonly used in nuclear physics and scattering theory [3]. It is usually written as

$$\exp(i\mathbf{k}\bullet\mathbf{r}) = 4\pi\sum_{\ell=0}^{\infty} i^{\ell} j_{\ell}(kr)\sum_{m=-\ell}^{\ell} Y_{\ell m}^{*}(\mathbf{k})Y_{\ell m}(\mathbf{r}) \qquad \text{(C1)}$$

where j_ℓ (z) is the spherical Bessel function of integer order ℓ, and *k* and *r* are the respective magnitudes of vector **k** and **r**. Because the argument of an exponential function must be dimensionless we deduce that vector **k** has the dimension of reciprocal length. No convergence condition accompanying this series expansion is reported. Equation C1 can easily be transformed[1] (see eq 10.47.12 in the NIST handbook[1]) to an equivalent expression for a *real* exponential argument, introducing the *modified* spherical Bessel function i_ℓ(z), given in eq C2,

$$i_{\ell}(z) = i^{-\ell} j_{\ell}(iz) \qquad \text{(C2)}$$

Setting *iz=kr* leads to *z= -ikr* and on to eq C3,

$$j_{\ell}(kr) = i^{\ell} i_{\ell}(-ikr) \qquad \text{(C3)}$$

Substituting eq C3 into eq C1 yields

$$\exp(i\mathbf{k}\bullet\mathbf{r}) = 4\pi\sum_{\ell=0}^{\infty} i^{2\ell} i_{\ell}(-ikr)\sum_{m=-\ell}^{\ell} Y_{\ell m}^{*}(\mathbf{k})Y_{\ell m}(\mathbf{r}) = 4\pi\sum_{\ell=0}^{\infty} i_{\ell}(ikr)\sum_{m=-\ell}^{\ell} Y_{\ell m}^{*}(\mathbf{k})Y_{\ell m}(\mathbf{r}) \qquad \text{(C4)}$$

where the parity relation $i_n(-x) = (-1)^n\, i_n(x)$ has been used. Equating the scalar i**k•r** to **k'•r'** yields ikrcosγ = i**k•r** = **k'•r'**= k'r'cosγ, which in turn enables the rewriting of eq C4 after dropping the dummy primes to eq C5, which has been numerically verified via a simple implementation,

$$\exp(\mathbf{k}\bullet\mathbf{r}) = 4\pi\sum_{\ell=0}^{\infty} i_{\ell}(kr)\sum_{m=-\ell}^{\ell} Y_{\ell m}^{*}(\mathbf{k})Y_{\ell m}(\mathbf{r}) \qquad \text{(C5)}$$

Using the well-known expression of eq C6,

$$P_{\ell}(\cos\gamma) = \frac{4\pi}{2\ell+1}\sum_{m=-\ell}^{\ell} Y_{\ell m}{}^{*}(\theta',\varphi')Y_{\ell m}(\theta,\varphi) \qquad \text{(C6)}$$

We can rewrite eq C6 as eq C7, which is equal to eq 7 of Kaufmann and Baumeister[2], by setting $\mathbf{k=r_1}$ and $\mathbf{r=r_2}$, and keeping in mind these vectors' different units, as discussed above,

---

[1] The Wolfram website mentions the condition z > 0 accompanying eq B2, quoting as a source page 633 in the book by Arfken and Weber, 1995 Edition. This condition is absent in formula 10.47.12 in the NIST handbook. This condition is problematic when *z= -ikr* because z > 0 is not obeyed because *kr* is real. We ignore this violation because we believe that the condition z > 0 is probably not necessary.

$$\exp(\mathbf{r}_1 \bullet \mathbf{r}_2) = \sum_{\ell=0}^{\infty} (2\ell+1)\, i_\ell(r_1 r_2) P_\ell(\cos\gamma) \qquad \text{(C7)}$$

This closes the proof.

For sake of completeness we note that Abramowitz and Stegun[4] lists eq 10.2.36,

$$\exp(z\cos\theta) = \sum_{n=0}^{\infty} (2n+1)\sqrt{\frac{\pi}{2z}} I_{n+1/2}(z) P_n(\cos\theta) \qquad \text{(C8)}$$

where the Bessel function of fractional order $I_{n+1/2}$ can be identified with the spherical Bessel functions while $\mathbf{r}_1 \bullet \mathbf{r}_2$ = $r_1 r_2 \cos\theta$ = $z\cos\theta$. Clearly, eq C8 and C7 cover the same identity. We also note that their equations under heading 10.2.13 confirm the explicit expressions for $i_\ell(x)$ on the relevant Mathematica web pages. Using a different notation the NIST handbook lists 10.60.8 as an alternative to eq C7.

Finally, an alternative treatment follows form another equation of Abramowitz and Stegun, eq 9.6.34, which avoids Legendre functions, and uses the modified Bessel function $I_n(z)$,

$$\exp(z\cos\theta) = I_0(z) + 2\sum_{n=1}^{\infty} I_n(z)\cos n\theta \qquad \text{(C9)}$$

Part D: Solving the radial integral of eq 18 (master equation) of the main text

We now return to the master equation, eq 18, which presents a radial and a double angular integral to be solved. The radial integral is tackled first, and can be written as

$$I_{rad}(\ell,S;\alpha,\beta,p)=\int_0^\beta dr\, r^{\lambda+\mu+\nu+L+2}\exp(-\alpha r^2)i_\ell(2\alpha Rr)=\int_0^\beta dr\, r^S\exp(-\alpha r^2)i_\ell(pr) \qquad \text{(D1)}$$

where $p = 2\alpha R$ and S = λ+μ+υ+L+2 simplify the notation of the integral. We repeat that λ, μ and υ are the respective powers of *x, y* and *z*, caused by the multiplication of the angular parts of the Gaussian primitives (p_x, p_y, p_z, d_{xy}, d_{xz}, ...). The variables ℓ and S are different to α, β and *p* because the latter are actually parameters, which do not determine the mathematical shape of the integral's solution.

The integral in eq D1 does not appear to have been solved based on ready inspection of published solved integrals. Therefore the integral had to be creatively reduced to known integrals. The open-ended character of the $i_\ell(r)$ function prompts writing the integral as a function of increasingly simpler integrals, in a recursive manner,

$$i_\ell(x)=i_{\ell-2}(x)-\left(\frac{2\ell-1}{x}\right)i_{\ell-1}(x) \qquad \text{(D2)}$$

Substitution of eq D2 into eq D1 leads to eq D3, where the parameters α, β and *p* have been dropped because they clutter the derivation,

$$\begin{aligned}
I(\ell,S)&=\int_0^\beta dr\, r^S\exp(-\alpha r^2)\left[i_{\ell-2}(pr)-\left(\frac{2\ell-1}{pr}\right)i_{\ell-1}(pr)\right]\\
I(\ell,S)&=\int_0^\beta dr\, r^S\exp(-\alpha r^2)i_{\ell-2}(pr)-\left(\frac{2\ell-1}{p}\right)\int_0^\beta dr\, r^{S-1}\exp(-\alpha r^2)i_{\ell-1}(pr) \qquad \text{(D3)}\\
I(\ell,S)&=I(\ell-2,S)-\left(\frac{2\ell-1}{p}\right)I(\ell-1,S-1)
\end{aligned}$$

This recursion formula contains a slight difficulty due to the mixed arguments of S and S-1. In other words, I(ℓ,S) cannot be obtained from a closed set of lower rank-ℓ I(ℓ,S) values. As a result it is not sufficient to calculate I(0,S) and I(1,S) in order to seed the recursion relation eq D3 and obtain any I(ℓ,σ). Instead, one also has to calculate a sequence of I(ℓ,S-1) integrals alongside I(ℓ,S). The best way to organise this recursion efficiently is to introduce the new variable σ and set it to S or S-1 as needed. Thus we are tasked with calculating I(0,σ) and I(1,σ), or

$$\begin{aligned}
I(0,\sigma)&=\int_0^\beta dr\, r^\sigma\exp(-\alpha r^2)i_0(pr)=\int_0^\beta dr\, r^\sigma\exp(-\alpha r^2)\frac{\sinh(pr)}{pr}=\frac{1}{2p}\int_0^\beta dr\, r^{\sigma-1}\exp(-\alpha r^2)\left[\exp(pr)-\exp(-pr)\right]\\
&=\frac{1}{2p}\left[\int_0^\beta dr\, r^{\sigma-1}\exp(-\alpha r^2+pr)-\int_0^\beta dr\, r^{\sigma-1}\exp(-\alpha r^2-pr)\right]=\frac{1}{2p}\left[I_f^+(\sigma-1)-I_f^-(\sigma-1)\right] \qquad \text{(D4)}
\end{aligned}$$

$$
\begin{aligned}
I(1,\sigma) &= \int_0^\beta dr\, r^\sigma \exp(-\alpha r^2) i_1(pr) = \int_0^\beta dr\, r^\sigma \exp(-\alpha r^2) \frac{(pr)\cosh(pr)-\sinh(pr)}{(pr)^2} \\
&= \frac{1}{2p}\int_0^\beta dr\, r^{\sigma-1}\exp(-\alpha r^2)\left[\exp(pr)+\exp(-pr)\right] - \frac{1}{2p^2}\int_0^\beta dr\, r^{\sigma-2}\exp(-\alpha r^2)\left[\exp(pr)-\exp(-pr)\right] \\
&= \frac{1}{2p}\int_0^\beta dr\, r^{\sigma-1}\exp(-\alpha r^2+pr) + \frac{1}{2p}\int_0^\beta dr\, r^{\sigma-1}\exp(-\alpha r^2-pr) - \frac{1}{2p^2}\int_0^\beta dr\, r^{\sigma-2}\exp(-\alpha r^2+pr) + \frac{1}{2p^2}\int_0^\beta dr\, r^{\sigma-2}\exp(-\alpha r^2-pr) \\
&= \frac{1}{2p} I_f^+(\sigma-1) + \frac{1}{2p} I_f^-(\sigma-1) - \frac{1}{2p^2} I_f^+(\sigma-2) + \frac{1}{2p^2} I_f^-(\sigma-2)
\end{aligned}
\qquad \text{(D5)}
$$

We now introduce *s* as the general argument of the fundamental integrals $I_f^+(s)$ and $I_f^-(s)$ where *s* can be σ-1 or σ-2. This integral is then defined as

$$I_f^\pm(s) = \int_0^\beta dr\, r^s \exp(-\alpha r^2 \pm pr) \qquad \text{(D6)}$$

According to both the book by Abramowitz and Stegun[4], and Mathematica's[5] Online Integral Calculator there *is no readily available closed expression for this integral.* However, one can proceed with the following indefinite integral (after correction of "dx" to "dt" in eq 10 in Section 2.33, p.108 in reference[6]) involving the *upper incomplete gamma function* (or gamma function of the second kind) Γ,

$$\int dx\, x^m \exp(-\beta x^n) = -\frac{\Gamma(\gamma,\beta x^n)}{n\beta^\gamma} = -\frac{1}{n\beta^\gamma}\int_{\beta x^n}^\infty dt\, t^{\gamma-1}e^{-t} \quad [\gamma = \frac{m+1}{n};\beta\neq 0; n\neq 0] \qquad \text{(D7)}$$

After setting n=2 and acknowledging that all conditional inequalities are satisfied, we make the following substitutions in order to use eq D7 to solve eq D6: β → α and m → s,

$$\int dx\, x^s \exp(-\alpha x^2) dx = -\frac{\Gamma(\gamma,\alpha x^2)}{2\alpha^\gamma} \quad \gamma = \frac{s+1}{2} \qquad \text{(D8)}$$

The final substitution involves the variable *x*, which is the most complicated. We set x = r+q, where *q* is a constant to be determined. Since *q* is a constant we can write that dx=dr. From x = r+q follows that $-\alpha x^2 = -\alpha(r^2+2rq+q^2) = -\alpha r^2 - 2\alpha q r - \alpha q^2$. The last term is a constant and hence can be treated as a prefactor to an integral. In order to recover $-\alpha r^2 \pm pr$ in the last right-hand side expression we demand that

$$\pm pr = -2\alpha q r \rightarrow q = \mp(p/2\alpha) \qquad \text{(D9)}$$

such that

$$-\alpha r^2 - 2\alpha q r = -\alpha x^2 + \alpha q^2 \rightarrow -\alpha r^2 \pm pr = -\alpha x^2 + \left(\frac{p^2}{4\alpha}\right) \qquad \text{(D10)}$$

such that

$$
\begin{aligned}
\int dr\, r^s \exp(-\alpha r^2 \pm pr) &= \exp(\frac{p^2}{4\alpha})\int dr\, (x \pm \frac{p}{2\alpha})^s \exp(-\alpha x^2) = \exp(\frac{p^2}{4\alpha})\int dr \left[\sum_{k=0}^{s}\binom{s}{k} x^k (\pm\frac{p}{2\alpha})^{s-k}\right]\exp(-\alpha x^2) \\
&= \exp(\frac{p^2}{4\alpha})\sum_{k=0}^{s}\binom{s}{k}(\pm\frac{p}{2\alpha})^{s-k}\int dr\, x^k \exp(-\alpha x^2) = -\exp(\frac{p^2}{4\alpha})\sum_{k=0}^{s}\binom{s}{k}(\pm\frac{p}{2\alpha})^{s-k}\frac{\Gamma\left(\frac{k+1}{2},\alpha x^2\right)}{2\alpha^{\left(\frac{k+1}{2}\right)}}
\end{aligned}
\qquad \text{(D11)}
$$

Taking into account the integration boundaries change from $x_1 = r_1 + q = 0 \mp (p/2\alpha)$ and $x_2 = \beta \mp (p/2\alpha)$ the *final result is*

$$I_f^{\pm}(s)=\int_0^{\beta} dr\, r^s \exp(-\alpha r^2 \pm pr)=\exp(\frac{p^2}{4\alpha})\sum_{k=0}^{s}\binom{s}{k}\frac{\left(\pm\frac{p}{2\alpha}\right)^{s-k}}{2\alpha^{\left(\frac{k+1}{2}\right)}}\left[\Gamma\left(\frac{k+1}{2},\alpha(\mp p/2\alpha)^2\right)-\Gamma\left(\frac{k+1}{2},\alpha[\beta\mp(p/2\alpha)]^2\right)\right] \quad \text{(D12)}$$

where Γ(a,x) is the upper incomplete gamma function (or of the second kind). If *a* is an integer then this functions returns essentially an exponential, while if *a* is a half-integer then the error function emerges. Note that the $\mp$ sign must be explicitly preserved when taking the square, because if a square root is taken of this square product it is important that the original sign is preserved. The result offered in D12 should be substituted into eqs D4 and D5, then allowing eq D3 to be evaluated, and thereby completing the task set by the opening equation, eq D1.

For sake of completeness we demonstrate plausibility of D12 by checking against explicit Mathematica formulae, starting with the first case where s=0 (such that k=0, and only a single term appears in the sum),

$$I_f^{\pm}(0)=\int_0^{\beta} dr \exp(-\alpha r^2 \pm pr)=\exp(\frac{p^2}{4\alpha})\frac{1}{2\alpha^{\left(\frac{1}{2}\right)}}\left[\Gamma\left(\frac{1}{2},\alpha(\mp p/2\alpha)^2\right)-\Gamma\left(\frac{1}{2},\alpha[\beta\mp(p/2\alpha)]^2\right)\right] \quad \text{(D13)}$$

In the Wikipedia entry on the incomplete gamma function we find

$$\Gamma(\frac{1}{2},x)=\sqrt{\pi}\, erfc\left(\sqrt{x}\right)=\sqrt{\pi}[1-erf\left(\sqrt{x}\right)] \quad \text{(D14)}$$

Hence

$$\begin{aligned} I_f^{\pm}(0)&=\int_0^{\beta} dr \exp(-\alpha r^2 \pm pr)=\frac{\exp(\frac{p^2}{4\alpha})}{2\sqrt{\alpha}}\left[\sqrt{\pi}[1-erf\left(\mp p/2\sqrt{\alpha}\right)]-\sqrt{\pi}[1-erf\left(\sqrt{\alpha}\beta\mp(p/2\sqrt{\alpha})\right)]\right] \\ &=\frac{\exp(\frac{p^2}{4\alpha})\sqrt{\pi}}{2\sqrt{\alpha}}\left[erf\left(\sqrt{\alpha}\beta\mp(p/2\sqrt{\alpha})\right)-erf\left(\mp p/2\sqrt{\alpha}\right)\right] \end{aligned} \quad \text{(D15)}$$

This is indeed the expression obtained by Mathematica for this specific case. We now check for the case of s=1.

$$\begin{aligned} I_f^{\pm}(1)&=\int_0^{\beta} dr\, r \exp(-\alpha r^2 \pm pr) \\ &=\exp(\frac{p^2}{4\alpha})\left\{\frac{\left(\pm\frac{p}{2\alpha}\right)}{2\sqrt{\alpha}}\left[\Gamma\left(\frac{1}{2},\alpha(\mp p/2\alpha)^2\right)-\Gamma\left(\frac{1}{2},\alpha[\beta\mp(p/2\alpha)]^2\right)\right]+\frac{1}{2\alpha}\left[\Gamma\left(1,\alpha(\mp p/2\alpha)^2\right)-\Gamma\left(1,\alpha[\beta\mp(p/2\alpha)]^2\right)\right]\right\} \\ &=\exp(\frac{p^2}{4\alpha})\left\{\frac{\pm p\sqrt{\pi}}{4\alpha^{3/2}}\left[-erf\left(\mp p/2\sqrt{\alpha}\right)+erf\left(\sqrt{\alpha}\beta\mp p/2\sqrt{\alpha}\right)\right]+\frac{1}{2\alpha}\left[\exp(-\alpha(\mp p/2\alpha)^2)-\exp\left(-\alpha[\beta\mp(p/2\alpha)]^2\right)\right]\right\} \\ &=\exp(\frac{p^2}{4\alpha})\left\{\frac{\pm p\sqrt{\pi}}{4\alpha^{3/2}}\left[-erf\left(\mp p/2\sqrt{\alpha}\right)+erf\left(\sqrt{\alpha}\beta\mp p/2\sqrt{\alpha}\right)\right]+\right\}+\frac{1}{2\alpha}\left[1-\exp\left(\beta[-\alpha\beta\pm p]\right)\right] \end{aligned} \quad \text{(D16)}$$

where we have used that $\Gamma(1,x)=e^{-x}$. Again we find that the last result is compatible with a specific and explicit expression obtained by Mathematica.

Finally, we comment on efficient implementation of the universal eq D12. One can make use of the more general equation for Γ, provided *s* is an integer. In this case no error functions appear (reserved for half-integer *s* values),

$$\Gamma(s,x)=(s-1)!e^{-x}\sum_{k=0}^{s-1}\frac{x^k}{k!}\quad s\in\mathbb{N}\qquad \text{(D17)}$$

A number of published indefinite integrals are useful to mention here, with each integration constant set to zero, for brevity. In particular, the following formula may be of use (Gradshteyn[6], Section 2.33, eq 11) for efficiency's sake:

$$\int x^m\exp(-\beta x^n)dx=-\frac{(\gamma-1)!}{n}\exp(-\beta x^n)\left[\sum_{k=0}^{\gamma-1}\frac{x^{nk}}{k!\beta^{\gamma-k}}\right]\quad \beta\neq 0,\ \gamma=\frac{m+1}{n}=1,2,3...\qquad \text{(D18)}$$

which in our case becomes (after setting n=2, β=α and m=s),

$$\int x^s\exp(-\alpha x^2)dx=-\frac{(\gamma-1)!}{2}\exp(-\alpha x^2)\left[\sum_{k=0}^{\gamma-1}\frac{x^{2k}}{k!\alpha^{\gamma-k}}\right]\quad \gamma=\frac{s+1}{2}\in\mathbb{N}\qquad \text{(D19)}$$

This equation is only valid if *s* is an odd integer. For s=1 we obtain

$$\int x\exp(-\alpha x^2)dx=-\frac{1}{2}\exp(-\alpha x^2)\left[\sum_{k=0}^{0}\frac{x^{2k}}{k!\alpha^{1-k}}\right]=-\frac{1}{2\alpha}\exp(-\alpha x^2)\qquad \text{(D20)}$$

which is compatible with a direct and explicit calculation with Mathematica, and for s=3 (γ=2) we obtain

$$\int x^3\exp(-\alpha x^2)dx=-\frac{1}{2}\exp(-\alpha x^2)\left[\sum_{k=0}^{1}\frac{x^{2k}}{k!\alpha^{2-k}}\right]=-\frac{1}{2}\exp(-\alpha x^2)\left[\frac{1}{\alpha^2}+\frac{x^2}{\alpha}\right]\qquad \text{(D21)}$$

which is also compatible with Mathematica and Gradshteyn[6] (Section 2.33, eq 13, p.109) (higher γ values are also listed there, in eqs 14 and 15). In summary, via recursion and breakdown into sufficiently elementary integrals, we have obtained a closed and general expression for the radial integral $I_{rad}(\ell,S;\alpha,\beta,p)$.

Part E: Solving the angular integral of eq 18 (master equation) of the main text

The angular integral in eq 18 of the main text is given again below for convenience, after dropping the index *i*,

$$I_{ang}(L,M,l,m,\lambda,\mu,\nu)=\int_0^{2\pi}\int_0^{\pi} d\theta\, d\varphi\, Y_{\ell,m}^*(\theta,\varphi)Y_{L,M}(\theta,\varphi)\sin^{\lambda+\mu+1}\theta\cos^{\nu}\theta\sin^{\mu}\varphi\cos^{\lambda}\varphi \quad \text{(E1)}$$

An appealing route to solve this integral is recasting eq E1 into Gaunt's integral (eq 34.3.22 in [1]),

$$\int_0^{2\pi}\int_0^{\pi} d\theta d\varphi \sin\theta\, Y_{\ell_1,m_1}(\theta,\varphi)Y_{\ell_2,m_2}(\theta,\varphi)Y_{\ell_3,m_3}(\theta,\varphi)=\left[\frac{(2l_1+1)(2l_2+1)(2l_3+1)}{4\pi}\right]^{1/2}\begin{pmatrix} l_1\, l_2\, l_3 \\ 0\,0\,0\end{pmatrix}\begin{pmatrix} l_1 & l_2 & l_3 \\ m_1 & m_2 & m_3\end{pmatrix} \quad \text{(E2)}$$

where the factors in brackets are *3j* symbols. However, we prefer to factorise the angular integral into a 1D integral in θ and one in φ. Using eqs A1 and E3,

$$Y_{\ell,m}^*(\theta,\varphi)=(-1)^m Y_{\ell,-m}(\theta,\varphi) \quad \text{(E3)}$$

we obtain,

$$Y_{\ell,m}^*(\theta,\varphi)=(-1)^m\left[\frac{(2\ell+1)}{4\pi}\frac{(\ell+m)!}{(\ell-m)!}\right]^{1/2}P_\ell^{-m}(\cos\theta)e^{-im\varphi}=(-1)^m\left[\frac{(2\ell+1)}{4\pi}\frac{(\ell+m)!}{(\ell-m)!}\right]^{1/2}(-1)^m\frac{(\ell-m)!}{(\ell+m)!}P_\ell^{m}(\cos\theta)e^{-im\varphi}$$
$$=\left[\frac{(2\ell+1)}{4\pi}\frac{(\ell-m)!}{(\ell+m)!}\right]^{1/2}P_\ell^{m}(\cos\theta)e^{-im\varphi} \quad \text{(E4)}$$

leading to

$$I_{ang}=\left[\frac{(2\ell+1)}{4\pi}\frac{(\ell-m)!}{(\ell+m)!}\right]^{1/2}\left[\frac{(2L+1)}{4\pi}\frac{(L-M)!}{(L+M)!}\right]^{1/2}I_\theta(L,M,l,m,\lambda,\mu,\nu)I_\varphi(M,m,\lambda,\mu)$$
$$=\left[\frac{(2\ell+1)}{4\pi}\frac{(\ell-m)!}{(\ell+m)!}\right]^{1/2}\left[\frac{(2L+1)}{4\pi}\frac{(L-M)!}{(L+M)!}\right]^{1/2}\int_0^{\pi}d\theta\, P_\ell^m(\cos\theta)P_L^M(\cos\theta)\sin^{\lambda+\mu+1}\theta\cos^{\nu}\theta\int_0^{2\pi}d\varphi\, e^{i(M-m)\varphi}\sin^{\mu}\varphi\cos^{\lambda}\varphi \quad \text{(E5)}$$

Using the formula of de Moivre we can transform the integral in φ, denoted I_φ, into

$$I_\varphi(M,m,\lambda,\mu)=\int_0^{2\pi}d\varphi\cos[(M-m)\varphi]\cos^{\mu}\varphi\sin^{\lambda}\varphi+i\int_0^{2\pi}d\varphi\sin[(M-m)\varphi]\cos^{\mu}\varphi\sin^{\lambda}\varphi \quad \text{(E6)}$$

This integral (either real or imaginary) is not listed in the classic book on integrals[6]. An original way of solving it is not to use de Moivre's formula but instead convert the cosine and sine functions to exponentials. Using the binomial theorem twice we obtain,

$$
\begin{aligned}
I_\varphi(M,m,\lambda,\mu) &= \int_0^{2\pi} d\varphi\, e^{i(M-m)\varphi}\cos^\lambda\varphi\sin^\mu\varphi = \int_0^{2\pi} d\varphi\, e^{i(M-m)\varphi}\left[\frac{1}{2}(e^{i\varphi}+e^{-i\varphi})\right]^\lambda\left[\frac{1}{2i}(e^{i\varphi}-e^{-i\varphi})\right]^\mu \\
&= \frac{1}{2^{\lambda+\mu}}\int_0^{2\pi} d\varphi\, e^{i(M-m)\varphi}\left[\sum_{j=0}^{\lambda}\binom{\lambda}{j}\left[e^{i\varphi}\right]^j\left[e^{-i\varphi}\right]^{\lambda-j}\right]\left[\sum_{k=0}^{\mu}\binom{\mu}{k}(-i)^\mu\left[e^{i\varphi}\right]^k\left[-e^{-i\varphi}\right]^{\mu-k}\right] \\
&= \frac{1}{2^{\lambda+\mu}}\sum_{k=0}^{\mu}\binom{\mu}{k}(-i)^\mu(-1)^{\mu-k}\sum_{j=0}^{\lambda}\binom{\lambda}{j}\int_0^{2\pi} d\varphi\, e^{i\varphi[M-m+j-(\lambda-j)+k-(\mu-k)]} \\
&= \frac{1}{2^{\lambda+\mu}}\sum_{k=0}^{p_y}\binom{\mu}{k}(-i)^\mu(-1)^{\mu-k}\sum_{j=0}^{\lambda}\binom{\lambda}{j}2\pi\delta_{2j+2k+M-m-\lambda-\mu,0}
\end{aligned} \qquad \text{(E7)}
$$

We note that the powers j and $\lambda-j$ could have been swapped in the application of the binomial theorem (and also k and $\mu-k$ of course). This means that the Kronecker delta imposes both the condition that 2(j+k)=λ+μ+m-M and that 2(j+k)=λ+μ+M-m, which can be summarised as 2(j+k)=λ+μ+|m-M|. This condition can only be satisfied if λ+μ+|m-M| is even. With this condition imposed, eq E7 becomes, after elimination of index j,

$$
I_\varphi(M,m,\lambda,\mu) = \frac{\pi}{2^{\lambda+\mu-1}}(-i)^\mu\sum_{k=0}^{\mu}(-1)^{\mu-k}\binom{\mu}{k}\binom{\lambda}{\frac{1}{2}(\lambda+\mu+m-M)-k} \quad \text{if } \lambda+\mu+|m-M| \text{ is even} \qquad \text{(E8)}
$$

It is very important to realise that I_φ vanishes if m > λ+μ+M. This is crucial in limiting the number of terms in eq. (18) of the main text. This inequality can be proven as follows.

First we state that λ, μ, r and k are all ≥ 0. We know that $\binom{j_1}{j_2}=0$ if $j_2 > j_1$. Thus, in order for $\binom{\lambda}{\frac{1}{2}(\lambda+\mu+m-M)-k}$ not to vanish we demand that ½(λ + μ + m − M) − k ≤ λ . The higher the value of k the most freedom there is to choose the other values and still obey this inequality. The maximum value that k can adopt is μ (see summation limit over index k). In that case we obtain that m ≤ M + λ + μ for the binomial expression *not* to vanish. In other words,

$$
I_\varphi(M, m > M+\lambda+\mu, \lambda, \mu) = 0 \qquad \text{(E9)}
$$

Using the fact that $\binom{n}{m}=0$ if m>n or if m<0, equations E8 and E9 has been successfully tested against the Wolfram online calculator for three cases when m=M: λ=μ=2; λ=2, μ=4; and λ=6, μ=4. They were also tested for the case of λ=μ=2 and when M–m=4. It is not obvious to immediately see that, if M=m, eq E8 leads to eq E10,

$$I_\varphi(M,M,\lambda,\mu)=\int_0^{2\pi} d\varphi\cos^\lambda\varphi\sin^\mu\varphi=\frac{2\Gamma\left(\frac{\lambda+1}{2}\right)\Gamma\left(\frac{\mu+1}{2}\right)}{\Gamma\left(\frac{\lambda+\mu+2}{2}\right)}\quad \text{if } \lambda \text{ and } \mu \text{ are both even} \qquad \text{(E10)}$$

a result simply taken (rather than computed from scratch) from processing four runs of the program Mathematica[5]. The alternative equation E11 fixes the condition inside the formula, or

$$I_\varphi(M,M,\lambda,\mu)=\int_0^{2\pi} d\varphi\cos^{2p}\varphi\sin^{2q}\varphi=\frac{2\Gamma(p+\frac{1}{2})\Gamma(q+\frac{1}{2})}{\Gamma(p+q+1)} \qquad \text{(E11)}$$

The shape of eq E11 indeed forces the powers of the cosine and sine function to be even.

As a quick digression we prove, using eq E11 and the orthogonality relation of associated Legendre polynomials, that we recover eq 19 when λ=μ=υ=0 is set in eq E1,

$$\begin{aligned}
I_{ang}(L,M,l,m,0,0,0)&=\int_0^{2\pi}\int_0^{\pi} d\theta d\varphi\sin\theta\, Y_{\ell m}^*(\theta,\varphi)Y_{LM}(\theta,\varphi)\\
&=\left[\frac{(2\ell+1)}{4\pi}\frac{(\ell-m)!}{(\ell+m)!}\right]^{1/2}\left[\frac{(2L+1)}{4\pi}\frac{(L-M)!}{(L+M)!}\right]^{1/2} I_\theta(L,M,l,m,0,0,0)I_\varphi(M,m,0,0)\\
&=\left[\frac{(2\ell+1)}{4\pi}\frac{(\ell-m)!}{(\ell+m)!}\right]^{1/2}\left[\frac{(2L+1)}{4\pi}\frac{(L-M)!}{(L+M)!}\right]^{1/2}\left[\int_0^{\pi} d\theta\sin\theta P_\ell^m(\cos\theta)P_L^M(\cos\theta)\right]\left[\frac{2\Gamma(\frac{1}{2})\Gamma(\frac{1}{2})}{\Gamma(1)}\delta_{mM}\right] \qquad \text{(E12)}\\
&=\left[\frac{(2\ell+1)}{4\pi}\frac{(\ell-m)!}{(\ell+m)!}\right]^{1/2}\left[\frac{(2L+1)}{4\pi}\frac{(L-M)!}{(L+M)!}\right]^{1/2}\left[\frac{2(\ell+m)!}{(2\ell+1)(\ell-m)!}\delta_{\ell L}\delta_{mM}\right]\left[2\pi\delta_{mM}\right]\\
&=\left[\frac{(2\ell+1)}{4\pi}\frac{(\ell-m)!}{(\ell+m)!}\frac{2(\ell+m)!}{(2\ell+1)(\ell-m)!}2\pi\right]\delta_{\ell L}\delta_{mM}\\
&=\delta_{\ell L}\delta_{mM}
\end{aligned}$$

where we used that Γ(1)=1 and $\Gamma\left(\frac{1}{2}\right)=\sqrt{\pi}$.

We now focus on the 1D integral in θ, which first appeared in eq E5, and is defined here again, having dropped the index *i*,

$$I_\theta(L,M,l,m,\lambda,\mu,\nu)=\int_0^{\pi} d\theta\, P_\ell^m(\cos\theta)P_L^M(\cos\theta)\sin^{\lambda+\mu+1}\theta\cos^\upsilon\theta \qquad \text{(E13)}$$

Again one can invoke a Gaunt-like expression but now with associated Legendre polynomials instead of spherical harmonics. Wikipedia lists a formula (the original one given by Gaunt in fact), which does not make use of Wigner 3j symbols but which involves an elaborate formula with many factorials, and several conditions. The challenge is then to cast the expression involving sine and cosine powers into an associated Legendre polynomial or a linear combination thereof. An appealing alternative would be to do the opposite and write out the associated Legendre polynomials as a function of sine and cosine powers. Once done

(even through mere listing rather than a general expression) eq E11 can be used to complete the integration. This route would allow sharing of source code. Yet another alternative is to use Erdélyi's formula listed as eq 14.13.1 in[1].

$$P_\ell^m(\cos\theta) = \frac{2^{m+1}(\sin\theta)^m}{\sqrt{\pi}} \sum_{k=0}^{\infty} \frac{\Gamma(\ell+m+k+1)}{\Gamma(\ell+k+\frac{3}{2})} \frac{(m+\frac{1}{2})_k}{k!} \sin[(\ell+m+2k+1)\theta] \qquad \text{(E14)}$$

A simple example (ℓ=m=0) already shows a drawback of this route,

$$P_0^0(\cos\theta) = 1 = \frac{2}{\sqrt{\pi}} \sum_{k=0}^{\infty} \frac{\Gamma(k+1)}{\Gamma(k+\frac{3}{2})} \frac{(\frac{1}{2})_k}{k!} \sin[(2k+1)\theta] = \frac{2}{\sqrt{\pi}} \left[\frac{2}{\sqrt{\pi}} \sin(\theta) + \frac{2}{3\sqrt{\pi}} \sin[3\theta] + \frac{1}{5}\frac{2}{\sqrt{\pi}} \sin[5\theta] + \ldots \right] \qquad \text{(E15)}$$

$$= \frac{4}{\pi}[\sin(\theta) + \frac{1}{3}\sin(3\theta) + \frac{1}{5}\sin(5\theta) + \ldots]$$

By virtue of 6.16.1 in the NIST handbook this identity is correct (allowing for the Gibbs phenomenon).

$$\sin(\theta) + \frac{1}{3}\sin(3\theta) + \frac{1}{5}\sin(5\theta) + \ldots = \frac{1}{4}\pi \quad for\, 0 < \theta < \pi; = 0 \; for\; x = 0 \; and \; -\frac{1}{4}\pi \; for -\pi < \theta < 0 \qquad \text{(E16)}$$

However, because the sum is not finite this identity is not attractive computationally, even less so because it will have to be applied twice while evaluating eq E13.

The route followed here takes as a starting point a formula given by Hobson[7]. This is attractive because the sum is finite and after double application the main integral $I_\theta(L,M,l,m,\lambda,\mu,\nu)$ will be reduced to one of pure sine and cosine powers.

$$P_\ell^m(\cos\theta) = \left(-\frac{1}{2}\right)^m (\ell+m)! \sum_{k=0}^{\left[\frac{\ell-m}{2}\right]} \left(-\frac{1}{4}\right)^k \frac{\cos^{l-m-2k}\theta \sin^{m+2k}\theta}{(\ell-m-2k)!(m+k)!k!} \qquad \text{(E17)}$$

where [ ] in the upper boundary of the summation marks the nearest integer function. Before testing this equation we points out that the original formula involved $P_{\ell m}(\cos\theta)$ but this was converted to $P_\ell^m(\cos\theta)$ by using $P_{lm}=(-1)^m P_l^m$ (see Part A). We successfully tested this formula on P_0^0, P_1^0, P_1^1, P_2^0, P_3^0, P_3^2 and P_3^3. To demonstrate the correctedness of eq E17 we now explicitly treat the case of P_4^2.

$$P_4^2(\cos\theta) = \left(-\frac{1}{2}\right)^2 6! \sum_{k=0}^{1} \left(-\frac{1}{4}\right)^k \frac{\cos^{2-2k}\theta \sin^{2+2k}\theta}{(2-2k)!(2+k)!k!} = 180\left[\frac{\cos^2\theta\sin^2\theta}{2!2!0!} - -\frac{1}{4}\frac{\sin^4\theta}{0!3!1!}\right] \qquad \text{(E18)}$$

$$= 45\sin^2\theta[\cos^2\theta - 1/6\sin^2\theta] = \frac{15}{2}(1-\cos^2\theta)\left[6\cos^2\theta - (1-\cos^2\theta)\right] = \frac{15}{2}(1-\cos^2\theta)(7\cos^2\theta - 1)$$

This result is compatible with the Wikipedia entry on Associated Legendre polynomials. We now substitute eq E17 in the master integral of eq E13,

$$I_\theta(L,M,l,m,\lambda,\mu,\nu)=\int_0^\pi d\theta\left(-\frac{1}{2}\right)^m\sum_{j=0}^{\left[\frac{\ell-m}{2}\right]}\left(-\frac{1}{4}\right)^j\frac{\cos^{l-m-2j}\theta\sin^{m+2j}\theta}{(\ell-m-2j)!(m+j)!j!}\left(-\frac{1}{2}\right)^M\times$$

$$\sum_{k=0}^{\left[\frac{L-M}{2}\right]}\left(-\frac{1}{4}\right)^k\frac{\cos^{L-M-2k}\theta\sin^{M+2k}\theta}{(L-M-2k)!(M+k)!k!}\sin^{\lambda+\mu+1}\theta\cos^{\upsilon}\theta \quad \text{(E19)}$$

$$=\left(-\frac{1}{2}\right)^{m+M}(\ell+m)!(L+M)!\sum_{j=0}^{\left[\frac{\ell-m}{2}\right]}\sum_{k=0}^{\left[\frac{L-M}{2}\right]}\left(-\frac{1}{4}\right)^{j+k}\frac{1}{(\ell-m-2j)!(m+j)!j!(L-M-2k)!(M+k)!k!}\times$$

$$\int_0^\pi d\theta\cos^{l-m-2j+L-M-2k+v}\theta\sin^{m+2j+M+2k+\lambda+\mu+1}\theta$$

The integral can be easily solved, essentially leading to the beta function, and is similar to eq E10 except for the different upper boundary,

$$I_{\cos/\sin}(a,b)=\int_0^\pi d\varphi\cos^a\varphi\sin^b\varphi=\left[1+(-1)^a\right]\frac{\Gamma\left(\frac{a+1}{2}\right)\Gamma\left(\frac{b+1}{2}\right)}{2\Gamma\left(\frac{a+b+2}{2}\right)} \quad \text{(E20)}$$

which clearly vanishes if the power of the cosine is odd, that is, *a* is odd,

$$a=l-m-2j+L-M-2k+v=(l-m+L-M+v)-2(j+k) \quad \text{(E21)}$$

In eq E21, the value of (l-m+L-M+υ) is parametrically fixed, as the task of solving the integral in eq E19 is set. Since 2(j+k) is even, (l-m+L-M+υ) must be odd for *a* to be odd. In other words, for each of the terms (j,k) in the double sum the power of the cosine will be odd and hence the whole integral will be zero:

$$if\ (l-m+L-M+v)\quad is\ odd\ then\ I_\theta=0 \quad \text{(E22)}$$

Part F: Solving for the G-type kinetic energy of the main text, eq 26

The contribution of the β sphere to the integrated kinetic energy, denoted $E_G(\beta)$, is defined as follows (repeating eq 26 of the main text exactly):

$$E_G(\beta) = \int_{\beta\ sphere} d\mathbf{r}\ E_G(\mathbf{r}) = \frac{1}{2}\sum_{p=1}^{n_{MO}} n_p \int_{\beta\ sphere} d\mathbf{r} \nabla \psi_p \bullet \nabla \psi_p \qquad \text{(F1)}$$

where all symbols in common with eq 3 in the main text mean the same. In the following derivation we proceed right away with Gaussian primitives of arbitrary angular momentum or

$$G_j(\mathbf{r}-\mathbf{R}_j;\alpha_j,\bar{l}_j,\bar{m}_j,\bar{n}_j) = \exp(-\alpha_j \left|\mathbf{r}-\mathbf{R}_j\right|^2)(x-X_j)^{\bar{l}_j}(y-Y_j)^{\bar{m}_j}(z-Z_j)^{\bar{n}_j} \qquad \text{(F2)}$$

using the name notation as in eq 8 of the main text. It is easy to prove that when the gradient, through its three partial derivatives in *x, y* and *z,* operates on a Gaussian primitive then new Gaussian primitives result:

$$\begin{aligned}
\frac{\partial}{\partial x} G_j(\mathbf{r}-\mathbf{R}_j;\alpha_j,\bar{l}_j,\bar{m}_j,\bar{n}_j) &= \bar{l}_j G_j(\mathbf{r}-\mathbf{R}_j;\alpha_j,\bar{l}_j-1,\bar{m}_j,\bar{n}_j) - 2\alpha_j G_j(\mathbf{r}-\mathbf{R}_j;\alpha_j,\bar{l}_j+1,\bar{m}_j,\bar{n}_j) \\
\frac{\partial}{\partial y} G_j(\mathbf{r}-\mathbf{R}_j;\alpha_j,\bar{l}_j,\bar{m}_j,\bar{n}_j) &= \bar{m}_j G_j(\mathbf{r}-\mathbf{R}_j;\alpha_j,\bar{l}_j,\bar{m}_j-1,\bar{n}_j) - 2\alpha_j G_j(\mathbf{r}-\mathbf{R}_j;\alpha_j,\bar{l}_j,\bar{m}_j+1,\bar{n}_j) \qquad \text{(F3)} \\
\frac{\partial}{\partial z} G_j(\mathbf{r}-\mathbf{R}_j;\alpha_j,\bar{l}_j,\bar{m}_j,\bar{n}_j) &= \bar{n}_j G_j(\mathbf{r}-\mathbf{R}_j;\alpha_j,\bar{l}_j,\bar{m}_j,\bar{n}_j-1) - 2\alpha_j G_j(\mathbf{r}-\mathbf{R}_j;\alpha_j,\bar{l}_j,\bar{m}_j,\bar{n}_j+1)
\end{aligned}$$

This recursion is a pleasant property of Gaussians, which ensures that previously solved integrals can be re-used. After introducing (q_s ; s=1,2,3) as corresponding to (*x, y, z*) we can write eq F1 as

$$\begin{aligned}
E_G(\beta) &= \frac{1}{2}\sum_{p=1}^{n_{MO}} n_p \int_\beta d\mathbf{r} \sum_{s=1}^{3} \frac{\partial}{\partial q_s}\left(\sum_{j=1}^{n_G} c_{jp} G_j(\mathbf{r}-\mathbf{R}_j;\alpha_j,\bar{l}_j,\bar{m}_j,\bar{n}_j)\right)\frac{\partial}{\partial q_s}\left(\sum_{k=1}^{n_G} c_{kp} G_k(\mathbf{r}-\mathbf{R}_k;\alpha_k,\bar{l}_k,\bar{m}_k,\bar{n}_k)\right) \\
&= \frac{1}{2}\sum_{p=1}^{n_{MO}} n_p \sum_{j=1}^{n_G}\sum_{k=1}^{n_G} c_{kp} c_{jp} \int_\beta d\mathbf{r} \left[\begin{array}{l} \left(\bar{l}_j G_j(\mathbf{r}-\mathbf{R}_j;\alpha_j,\bar{l}_j-1,\bar{m}_j,\bar{n}_j) - 2\alpha_j G_j(\mathbf{r}-\mathbf{R}_j;\alpha_j,\bar{l}_j+1,\bar{m}_j,\bar{n}_j)\right)\times \\ \left(\bar{l}_k G_k(\mathbf{r}-\mathbf{R}_k;\alpha_k,\bar{l}_k-1,\bar{m}_k,\bar{n}_k) - 2\alpha_k G_k(\mathbf{r}-\mathbf{R}_k;\alpha_k,\bar{l}_k+1,\bar{m}_k,\bar{n}_k)\right) \\ +\ equivalent\ terms\ for\ q_2(or\ y)\ and\ q_3(or\ z). \end{array}\right] \qquad \text{(F4)} \\
&= \frac{1}{2}\sum_{p=1}^{n_{MO}} n_p \sum_{j=1}^{n_G}\sum_{k=1}^{n_G} c_{kp} c_{jp} \int_\beta d\mathbf{r} \sum_{r=1}^{12} F_{jk,r} G_{jk}(\mathbf{r}-\mathbf{R}_{jk};\alpha_{jk},\bar{l}_{jk,r},\bar{m}_{jk,r},\bar{n}_{jk,r}) \\
&= \frac{1}{2}\sum_{p=1}^{n_{MO}} n_p \sum_{j=1}^{n_G}\sum_{k=1}^{n_G} c_{kp} c_{jp} E_{G,jk}(\beta)
\end{aligned}$$

In contrast to the expressions for $Q_{LM,jk}$, an extra prefactor, denoted $F_{jk,r}$, occurs in eq F5, which can take values such as $\bar{l}_j\bar{l}_k$, $-2\bar{l}_j\alpha_k$ or $4\alpha_j\alpha_k$, for example. The powers $\bar{l}_r,\bar{m}_r,\bar{n}_r$ vary from term to term (in the sum over index *r*) and carry an imprint of the differentiation caused by the gradient operator.

In the current absence of a source code implementation it is not useful to write out the 12 (= 4x3) terms appearing in the square brackets in eq F5. Instead, it is more important to show how the kinetic energy contribution $E_{G,jk}(\beta)$ "makes contact" with the previously solved integrals, thus proving that an implementation will benefit from those integrals via a chain of equations. In this spirit, we just reiterate and thereby clarify that the explicit product in the square bracket of eq F4 leads to, after using eq 4 of the main text,

$$\overline{l}_j \overline{l}_k K_{jk} \exp(-\alpha_{jk} \left|\mathbf{r}-\mathbf{R}_{jk}\right|^2)(x-X_j)^{\overline{l}_j-1}(y-Y_j)^{\overline{m}_j}(z-Z_j)^{\overline{n}_j}(x-X_k)^{\overline{l}_k-1}(y-Y_k)^{\overline{m}_k}(z-Z_k)^{\overline{n}_k} + 3\ other\ terms \qquad \text{(F5)}$$

The "3 other terms" have in common the general shape of the product Gaussian primitive ($G_{jk}(\mathbf{r} - \mathbf{R}_{jk})$), the only difference with previous expressions being the prefactors $F_{jk,r}$.

We are now in a position to follow the logic from eq 9 in main text onwards leading to eq 11, where the binomial expression was used to work out the products in eq F5. The only difference now lies in the exact powers of the *x, y* and *z* part (i.e. caused by shifts, from $\overline{l}_j$ to $\overline{l}_j - 1$, for example). This difference affects the $f_{\lambda x}$ –type functions, which now receive a subscript *r* in order to express these shifts in powers. Thus, the equivalent of eq 11, which formulates the key integral for $Q_{LM,jk}$, now becomes

$$E_{G,jk}(\beta) = K_{jk} \sum_{r=1}^{12} F_{jk,r} \sum_{\lambda=0} \sum_{\mu=0} \sum_{\nu=0} f_{\lambda x,r} f_{\mu y,r} f_{\nu z,r} \int_{\beta} d\mathbf{r}\ \exp(-\alpha_{jk} \left|\mathbf{r}-\mathbf{R}_{jk}\right|^2) x^{\lambda} y^{\mu} z^{\nu} \qquad \text{(F6)}$$

This equation concludes the proof that the G-type kinetic energy can be calculated by closed expressions because the integral in eq F6 is a special case (i.e. L=M=0 in $R_{LM}(\mathbf{r})$) of the integral in eq 11. Hence the treatment of the integral in eq F6 is identical to that in eq 11.

References

(1) *NIST Handbook of Mathematical Functions*; Olver, F. W. J.; Lozier, D. W.; Boisvert, R. F.; Clark, C. W., Eds.; Cambridge University Press: Cambridge, Great Britain, 2010.

(2) Kaufmann, K.; Baumeister, W. *J.Phys.B.* **1989**, *22*, 1.

(3) Newton, R. G. *Scattering Theory of Particles and Waves*; McGraw-Hill: New York, USA, 2002.

(4) Abramowitz, M.; Stegun, I. A. *Handbook of Mathematical Functions, 9th Ed.*; Dover: New York, USA, 1971.

(5) Mathematica Wolfram Research, Inc., Champaign, IL, USA 2013.

(6) Gradshteyn, I. S.; Ryzhik, I. M. *Table of Integrals, Series and Products, 7th Ed.*; Academic Press Inc, London, Great Britain, 2007.

(7) Hobson, E. W. *The Theory of Spherical and Ellipsiodal Harmonics* Chelsea, New York, USA, 1955.
